# The Intercontinental phylogeography of neustonic daphniids

**DOI:** 10.1038/s41598-020-58743-8

**Published:** 2020-02-04

**Authors:** Derek J. Taylor, Sandra J. Connelly, Alexey A. Kotov

**Affiliations:** 1 Department of Biological Sciences, The State University of New York at Buffalo, Buffalo, NY 14260 USA; 2 Thomas H. Gosnell School of Life Sciences, Rochester Institute of Technology, Rochester, NY USA; 3 A. N. Severtsov Institute of Ecology and Evolution, Leninsky Prospect 33, 119071 Moscow, Russia

**Keywords:** Biodiversity, Biogeography, Freshwater ecology, Evolutionary genetics, Taxonomy, Evolution, Limnology

## Abstract

Organisms that live at the freshwater surface layer (the neuston) occupy a high energy habitat that is threatened by human activities. Daphniids of the genera *Scapholeberis* and *Megafenestra* are adapted to the neuston but are poorly studied for biogeography and diversity. Here we assess the global phylogeography of neustonic daphniids. We obtained 402 new multigene sequences from the 12S rRNA, 16S rRNA, and tRNA (val) regions of the mitochondrial genomes of daphniids from 186 global sites. We assessed the intercontinental origins and boundaries of mitochondrial lineages and the relative rates of evolution in neustonic and planktonic daphniids. We identified 17 divergent lineages in the neustonic daphniids that were associated with biogeographic regions. Six of these lineages had intercontinental ranges – four of these were Transberingian. Patagonian populations of *Scapholeberis rammneri* were monophyletic and nested within a closely related clade of western North American haplotypes, suggesting an introduction from the Western Nearctic to South America. The Eastern Palearctic was more diverse than other regions, containing eight of the major lineages detected in the Scapholeberinae. The Genus *Scapholeberis* had high levels of divergence compared to non-neustonic daphniids. Neustonic daphniids have more divergent biogeographic lineages than previously appreciated.

## Introduction

Freshwater habitats are currently threatened by global change^1–4^. However, we still know little about the biogeography and diversity of their component ecosystems. One of the least studied and most threatened freshwater components is the neuston – organisms that live in the surface film. As the surface interface lacks a refuge from ultraviolet radiation, the neuston is an extreme habitat for transparent organisms. One reason for the lack of study is that sampling the neuston requires collection at the air-water interface. Historical efforts often targeted the plankton–benthic and neustonic organisms were often considered the limnological “bycatch”. As the freshwater neuston is a required habitat for the immature stages of mosquitoes^5^, humans have targeted the layer for widespread disruption (through the application of surfactants, oils, and polystyrene beads). Finally, neustonic organisms are often less than 1 mm in size, complicating genetic studies. The current lack of biogeographical knowledge for the neuston hinders a detailed understanding of the biotic implications of ongoing changes and threats for freshwater.

Some arthropods, such as daphniids of the Subfamily Scapholeberinae Dumont et Pensaert have adult stages that are adapted to the neuston. The Subfamily Scapholeberinae contains two genera that are hyponeustonic, with *Megafenestra* Dumont et Pensaert (two known species) being more planktonic than *Scapholeberis* Schoedler. *Scapholeberis* (with eight proposed species and one subspecies^6^), spends much of its time attached to the lower edge of the surface film (the hyponeuston). Neustonic daphniids are also unusual among temperate daphniids in possessing a heavily melanized, flattened ventral margin. Several authors have hypothesized that the pigmentation is an adaptation to the neuston, which may mitigate damage from the intense UV radiation at the water-surface interface^6^.

Most of the known diversity of Scapholeberinae is believed to be temperate and Holarctic^6^. However, large parts of the Holarctic, Africa, and South America remain poorly sampled^7,8^. Additional taxonomic confusion has been attributed to the presence of presumptive defensive structures such as a horn-like structure on the head and mucro spine on the postero-ventral valve corner^9,10^. There is historical confusion in the literature concerning several species that produce horns such as *S. kingii*, *S. mucronata*, and *S. rammneri*^11^. Dumont and Pensaert (1983) used additional morphological characters to infer a phylogeny for the genus *Scapholeberis* whereby *S. microcephala* is basal to the sister groups *S. mucronata* and the “*S. kingii* group” (which itself contains a New World and an Old World clade). The most recently described species of the subfamily is *Scapholeberis duranguensis* Quiroz-Vazquez et Elias-Gutierrez from Northern Mexico^8^. Several species have now been recorded on multiple continents (e.g., *S. mucronata*, *S. rammneri*, *S. armata*, and *S. kingii*). However, it is presently difficult to determine if disjunct distributions (often transcontinental) result from recent introductions, cryptic sister taxa, or taxonomic confusion. Moreover, the Scapholeberinae are among the only cladocerans that lack an historical subfossil record in the sediments^12^.

DNA sequencing efforts for the Scapholeberinae have been minimal, with a focus on a single specimen or populations. The position of the Scapholeberinae is still unresolved as a result of weak phylogenetic support^13^, or conflicting signals in multigene studies^14,15^. Cornetti *et al*.^14^ suggested that the conflict in their data may be due to the independent evolution of the neustonic habit in *Scapholeberis* and *Megafenestra*. They also proposed that the 12S and 16S rRNA gene regions should be the “first choice” of genetic marker over mitochondrial protein-coding regions for the study of daphniids. Their reasoning is based on the finding that daphniid phylogenies using mitochondrial rRNA gene regions show much more agreement with the phylogenies from 636 nuclear loci than do the phylogenies using mitochondrial protein coding regions (this includes a daphniid tree based on all 13 protein coding genes). The main disagreements in the daphniid phylogenies involve the placement of *Scapholeberis mucronata*. With the mitochondrial protein coding genes, *Scapholeberis* (represented by a single clone) is placed in its traditional position (based on morphology) as a sister group to *Megafenestra*. However, the nDNA gene analyses place *Scapholeberis* as basal to all other daphniid genera (including *Megafenestra*). The authors conclude that more taxonomic sampling (beyond the genus *Daphnia*) will be necessary to address the incongruence.

Benthic and planktonic cladocerans have, in general, shown pronounced regionalism at the Holarctic scale with clear genetic influences of Pleistocene glaciation and increased refugial diversity^16–20^. Within the Holarctic, the Eastern Palearctic is often found to be a region of high cladoceran diversity, an observation attributed to reduced extinction during the Pleistocene and to the contact of expanding refugial lineages^21^. Several cladocerans have become established and successful transcontinental invaders^22–27^.

The biogeographic and evolutionary consequences of the neustonic habitat for daphniids remain unknown. Increases in rates of evolution associated with high UV radiation environments have been proposed to play a role in increased speciation and species-energy relationships^28^. UV radiation has the potential to damage freshwater zooplankton^29^. Because UV radiation exposure attenuates in freshwater with depth, the exposure to UV radiation (especially UV–B) is greater for obligately neustonic daphniids compared to the UV exposure of benthic or planktonic daphniids. UV radiation damage to nucleic acids is sequence specific with the most frequently affected dinucleotide being TT^30,31^. Recently it has become clear that neighboring nucleotides to TT also promote or inhibit lesion formation. The largest effect is the contrast between trinucleotides that promote lesions (TTA, TTT) and those that inhibit lesions for TT(TTC, TTG)^31^. Note that both purines and pyrimidines can enhance or inhibit lesion formation depending on the sequence context. Daphniids have adaptations to modestly increased UV environments involving photolesion repair enzymes and UV-filtering pigments such as melanin^32^. However, in high energy habitats, genomes with fewer photolesion targets would presumably require less repair and be favored by selection (i.e., a UVR-adapted genome hypothesis). Planktonic daphniids from high energy habitats (saline waters) have moderately increased evolutionary rates, but the trinucleotide signatures with strong UV-lesion effects remain unexamined^33^. It is also unknown if the more extreme UV exposure of neustonic daphniids has affected the salient trinucleotide compositions (TTA, TTT) of AT-rich mitochondrial DNA.

In the present study, we aimed to conduct global sampling and genetic analyses to assess multicontinental biogeographic connections in the Scapholeberinae. We found at least six species/subspecies have geographic clades that traverse continental boundaries. We provide evidence of non-anthropogenic immigration of *S. rammneri* into South America from North America. Transberingian connections were apparent for four species. However, several named species also have regional novel geographic clades, including species in the poorly studied genus *Megafenestra*. Our results suggest that the cladoceran neuston has greater diversity, biogeographic structure and mitochondrial evolutionary rate variation than previously appreciated.

## Results

### Phylogeny

**Figure 1:**
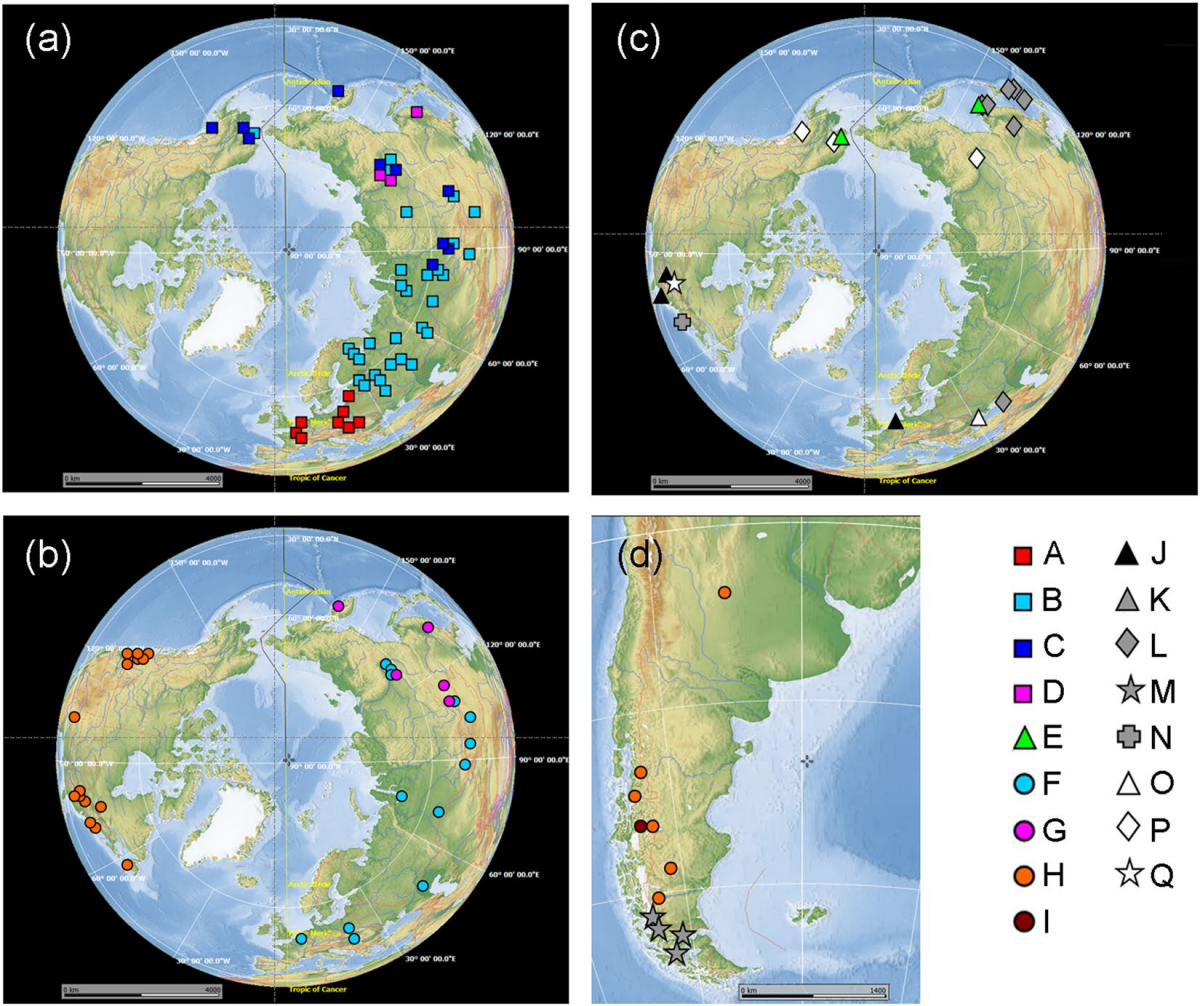
Map of populations studied for the phylogeography of neustonic daphniids (*Scapholeberis* and *Megafenestra*). Symbols correspond to mitochondrial clades (see Fig. 2) (**a**) Locations for specimens of the *S. mucronata* species group; (**b**) Populations of *Megafenestra* (clear symbols), *S. microcephala*, the *S. kingii* complex, and the *S. armata* complex; (**c**) populations of *S. rammneri* in the Nearctic; and (**d**) populations of *S. rammneri* and *S. spinifera* in South America. The base maps are from the public domain atlas in the desktop app, Marble 2.2.20 (http://edu.kde.org/marble). Symbols were placed manually in Adobe Photoshop using the output from DIVA-GIS 7.5 (https://www.diva-gis.org/) as a guide.

**Figure 2:**
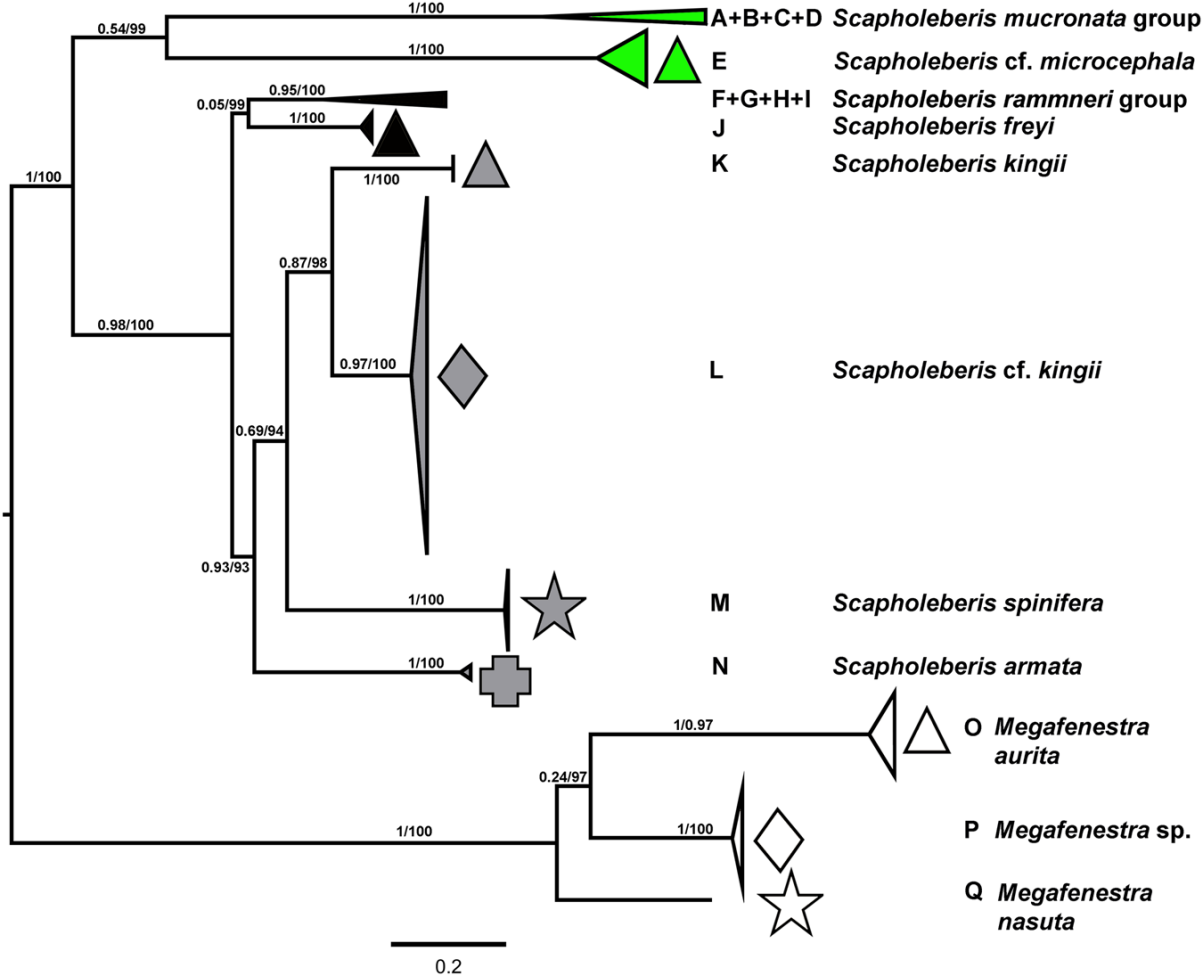
Maximum likelihood mitochondrial phylogeny of neustonic daphniids (*Scapholeberis* and *Megafenestra*). Bold letters (**A–Q**) indicate geographic clades. Numbers at the nodes indicate approximate likelihood ratio tests (aLRTs)/nonparametric bootstrap support. Colours represent deeper clades in the Scapholeberinae. The tree is midpoint rooted. See Appendix S2 for an outgroup rooted tree and a tree expanded to show individual sequences.

After trimming, the alignment length with all taxa was 1153 nucleotides (a second Scapholeberinae -only alignment was 1074 bases in length). A BLAST comparison yielded a 100% coverage to a contig in a recent assembly of *Scapholeberis* from eastern North America (contig c29081_g2_i1;^15^). This contig (which also contained an ND1 ORF) was almost identical in sequence to eastern North American sequences from the present study (minimum p-distance = 0.005). The published mitochondrial genome of *Scapholeberis* cf. *mucronata*^14^ also had a complete segment matching the present sequences (see below). We detected 17 mitochondrial clades of Scapholeberinae (Figs. 1, 2, and S1). Three of these clades were in the genus *Megafenestra* – two corresponded to the known named species (*M. aurita* and *M. nasuta*). The newly discovered clade of *Megafenestra* had a Transberingian range and could not be assigned morphologically into existing taxa. Only two sequences within the genus *Scapholeberis* had significant base compositional heterogeneity with a Scapholeberinae-only alignment (see Fig. 1). The optimal substitution model for the Scapholeberinae was Tamura-Nei + F + I + G, which uses three substitution parameters. The application of the alignment noise-filtering algorithm Gblocks, removed only 11% of sites for the Scapholeberinae alignment.

**Figure 3:**
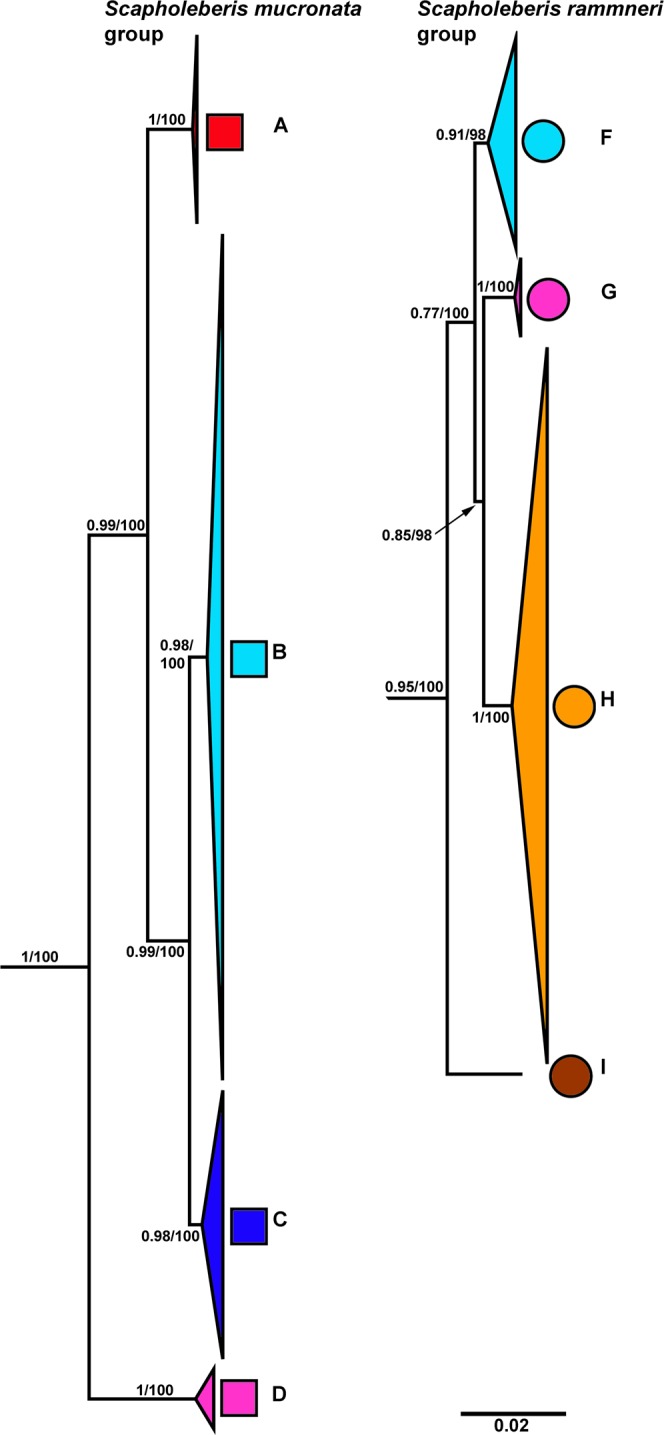
Expanded regions of the Maximum likelihood mitochondrial phylogeny in Fig. 2. Geographic clades of the *Scapholeberis mucronata* group (left) and the *Scapholeberis rammneri* group (right) are depicted. Bold letters (**A–D**,**F–I**) indicate geographic clade names from Fig. 2. Numbers at the nodes indicate approximate likelihood ratio tests (aLRTs)/nonparametric bootstrap support. See Appendix S2 for an outgroup rooted tree and a tree expanded to show individual sequences.

*Scapholeberis* had a substantial division with *S. mucronata*/*S*. cf. *microcephala* in one group and *S. rammneri*/*S. kingii*/*S. spinifera*/*S. armata* in the other group. *S. mucronata* had four main geographic clades (A + B + C + D; Fig. 3). Clade A (*S. mucronata s. str*.) was detected only in Western and Central Europe; clade B was detected from European Russia to Yakutia and Alaska (i.e. trans-Beringian); clade C was found from Western Siberia through Eastern Siberia, the Kamchatka Peninsula, and Western Alaska. Clade D was found in the far eastern Palearctic, overlapping with B, and C. *S*. cf. *microcephala* had a trans-Beringian distribution area, it was detected from a single locality on Sakhalin Island and from a single locality near Council, Alaska.

The *S. rammneri* species group included clades (F + G + H + I + J; Figs. 3, S1). Clade F (*S. rammneri s.str*.) was widely distributed in Northern Eurasia, but was not detected in the Beringian region; clade G was present only in Eastern Siberia and the Far East of Russia; clade H was widely distributed in North America, and also detected from several localities in Patagonia; clade I was detected only in a single lake is southern South America (Patagonian Chile). Clade J (*S. freyi*) was detected at two localities in the eastern portion of North America and a genomic sequence from Europe (ENA: LS991522.1). A BLAST search with the genomic sequence as a query (LS991522.1) also returned *S. freyi* (KU315490.1 from Brazil) as the closest match at 92% similarity for the cytochrome oxidase subunit 1 gene.

Grouping with *S. rammneri* were clades K (*Scapholeberis kingii s. str*.), L (*S*. cf. *kingii*), M (*S. spinifera*) and N (*S*. cf. *armata*). *Scapholeberis kingii s. str*. (Clade K) was detected only in the type location continent of Australia; clade L was present in the Far East of Russia (including Sakhalin Island), Japan, and a single population in the southernmost part of European Russia; clade N was represented by a single population from the Atlantic Coast of North America.

### Support and network analysis

**Figure 4:**
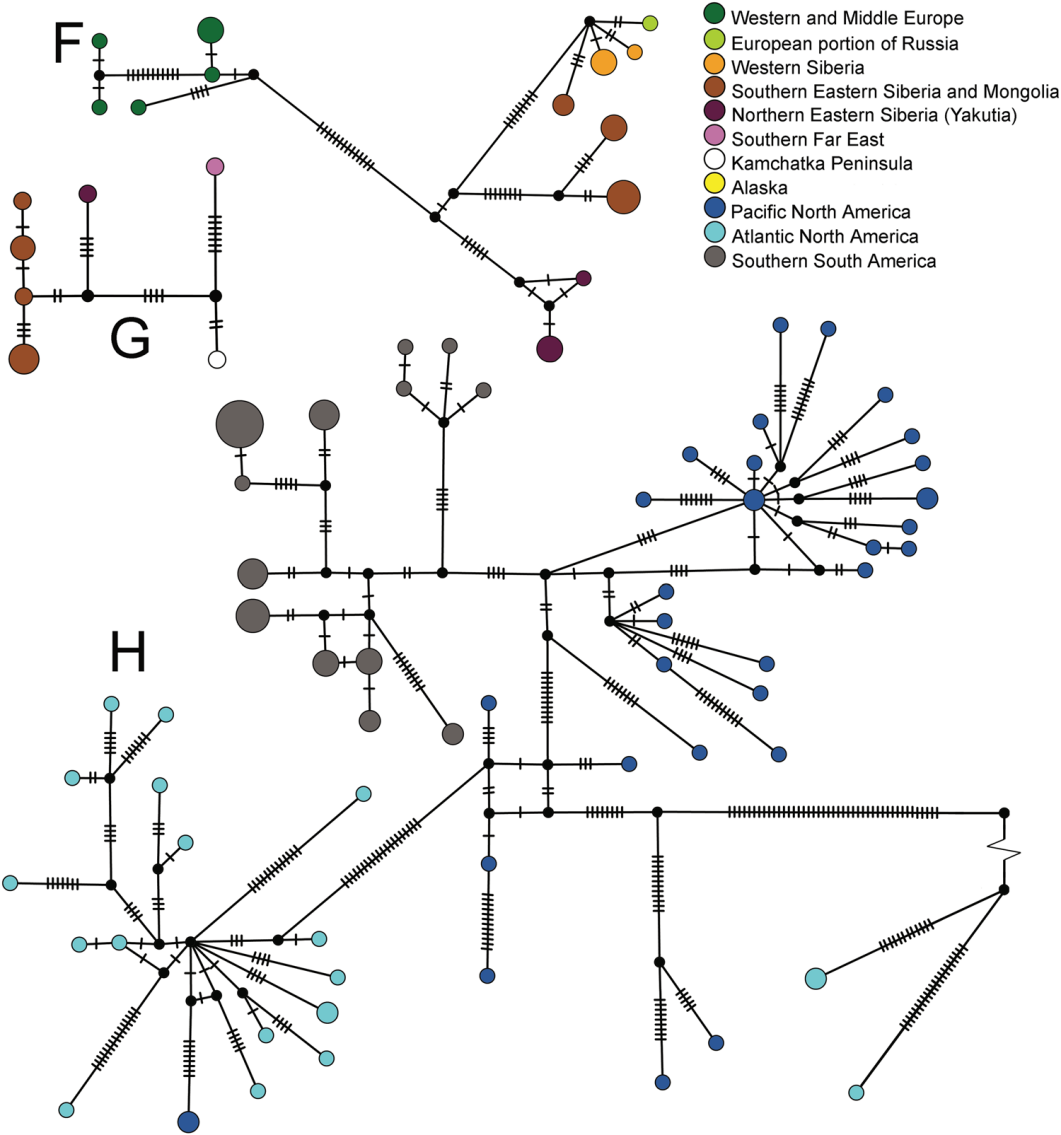
Median-joining haplotype networks of mitochondrial clades for neustonic daphniids in the *Scapholeberis rammneri* group. Bold letters indicate geographic clade names provided in Fig. 2. Pie diameter indicates the frequency of the haplotype and substitutions among haplotypes are depicted by hash marks.

**Figure 5:**
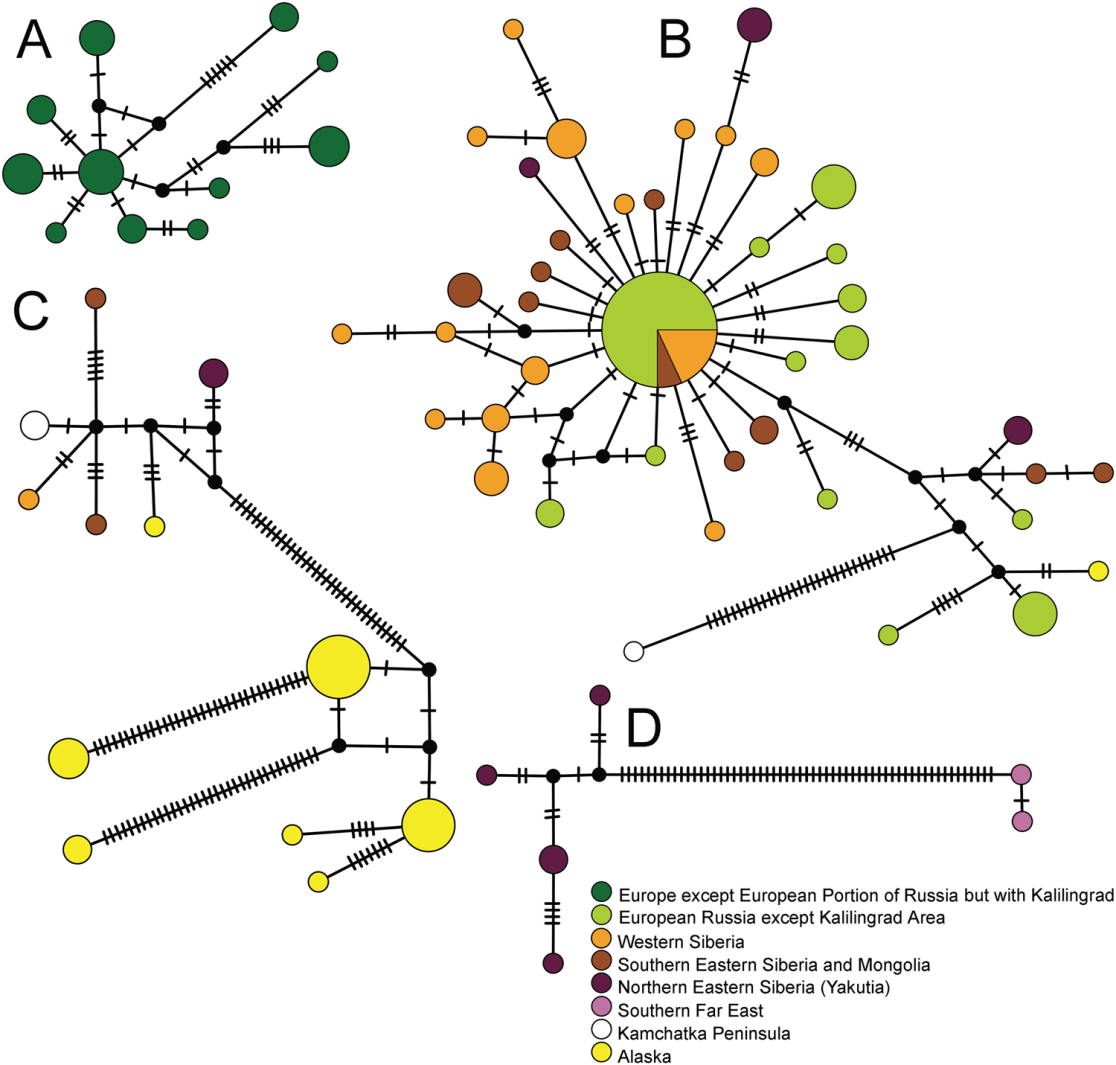
Median-joining haplotype networks of mitochondrial clades for neustonic daphniids in the *Scapholeberis mucronata* group. Bold letters indicate geographic clades (see Fig. 2). Pie diameter indicates the frequency of the haplotype and substitutions among haplotypes are depicted by hash marks.

The divergent geographic clades (lettered) had strong bootstrap support (>95/95; Fig. S2). As expected, the transfer bootstrap support was higher than the nonparametric bootstrap for several deeper nodes in this large tree. The subclade of Patagonian specimens of *S. rammneri* grouped within western North American populations in Clade H (Fig. S2). Although network analysis revealed substantial haplotype diversity (Fig. 4), there was strong support for monophyly of the Patagonian *S. rammneri* (Fig. S2). The South American clade of *S. rammneri* had a haplotype diversity (Hd) of 0.89331 and an average number of differences of K: 8.70839. Both measures are less than those of the related clade of western North American *S. rammneri*: Hd = 1.0 and K = 10.23715. Note that we did also detect a more divergent lineage in western Patagonia (Clade I) that is related to *S. rammneri*. The average number of differences with both clades was Kt = 11.49180. The western Palearctic (A) and central Palearctic clades (B) of *S. mucronata* appeared to have a star-like network shape of closely related haplotypes, while the far Eastern and Beringian haplotype networks were an amalgam of local but divergent haplotypes (Fig. 5).

### Timetree and UVR- relevant trinucleotide targets

**Figure 6:**
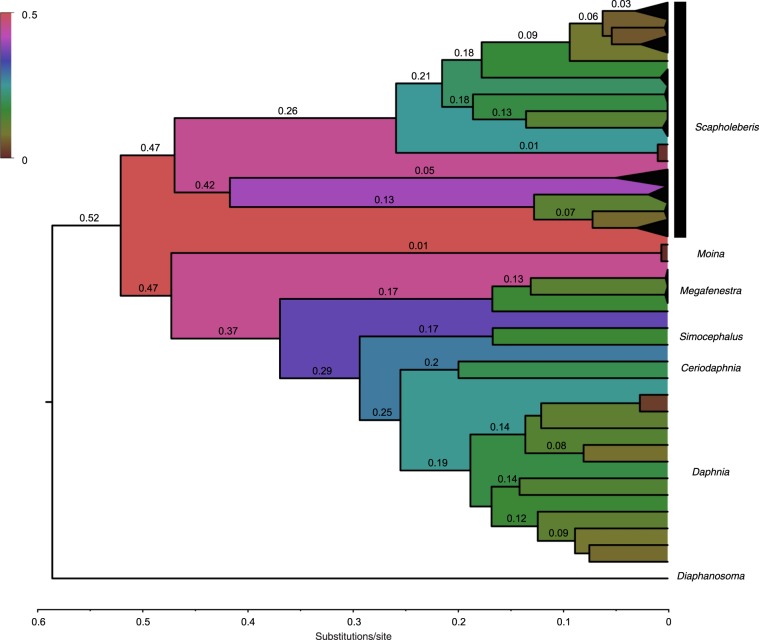
Time tree of mitochondrial sequence variation in neustonic (*Scapholeberis* and *Megafenestra*) and non-neustonic daphniids. The graph shows the relative rates of genetic divergence in substitutions/site (numbers on axis, branches and colors depict the same values) using a maximum likelihood tree as input.

**Figure 7:**
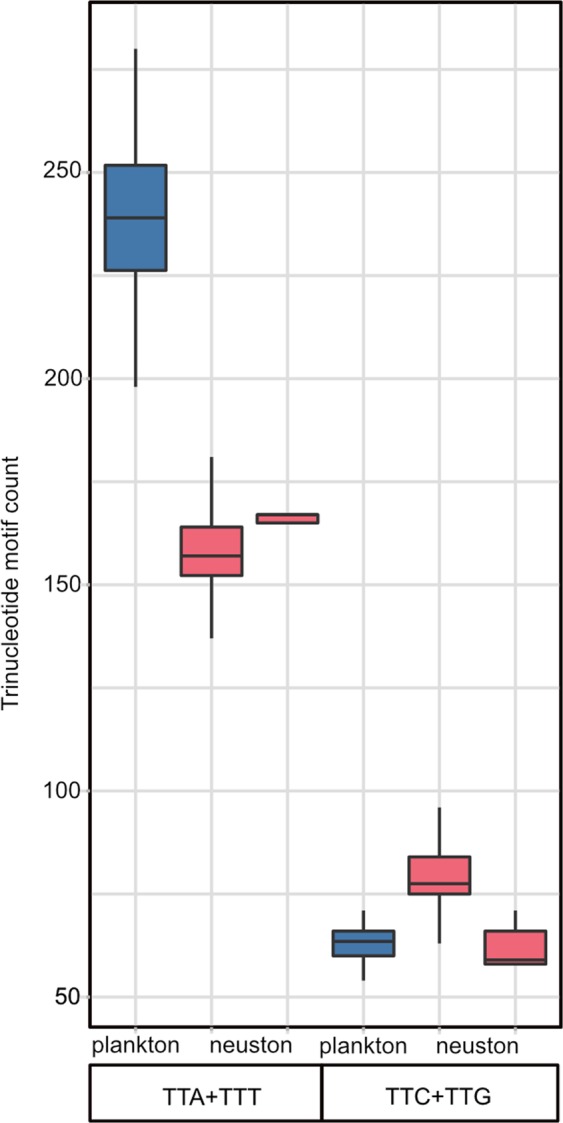
Box and whiskers plots comparing counts of trinucleotide motifs in cladoceran crustacean DNA sequences from the rRNA gene region of mitochondrial genomes. The motifs from the plus and negative strands were summed to attain the final counts. The motifs on the left (TTA/TTT) have been shown to promote UV radiation (UVR) photolesion formation while those on the right (TTC/TTG) are noted for lacking photolesion formation. Box plots are colored by habitat with the blue boxes representing planktonic daphniids and moinids (low UVR habitat, N = 22); and pink boxes (high UVR neustonic habitats) representing sequences from specimens of *Scapholeberis* (N = 358; left) and *Megafenestra* (N = 13; right). Sequence and specimen details are available in the Supplementary Data.

The phylogeny failed to indicate monophyly of the Scapholeberinae. *Megafenestra* grouped at the base of the non-scapholeberine daphniids, while *Scapholeberis* grouped basal to *Moina* and daphniids. To compare relative evolutionary rates, we estimated a Timetree (Fig. 6). The graph indicates that there is as much evolution (0.47 substitutions per site in the ML Timetree) in the mitochondrial genome detected within the genus *Scapholeberis* as between families of Cladocera (Daphniidae and Moinidae). Moreover, the evolutionary divergences within the genus *Scapholeberis* are high throughout the graph (as they are in the other ML trees estimated with differing models; Figs. S1 and S3). The divergence among several species of *Scapholeberis* is greater than the several among-genus contrasts in the planktonic Daphniidae. For example, *Scapholeberis* contains nearly twice the divergence of the *Ceriodaphnia*/*Daphnia* split. The discordance of mitochondrial evolution is great when it is considered that *S. rammneri* and *S. mucronata* are often confused for one another based on morphology. Although some of the clades of *Scapholeberis* grouped with other daphniids, the GHOST model of evolution (designed to address rate variation across lineages or heterotachy) also failed to recover a monophyletic Scapholeberinae (Fig. S3). Moreover, the long branches within the genus *Scapholeberis* are still apparent in the heterotachy tree. Counts of photolesion-promoting trinucleotides (TTA/TTT) were markedly reduced in neustonic daphniids (*Scaphobeleris* and *Megafenestra*) compared to the same counts in planktonic daphniids and moinids (Fig. 7). The lowest planktonic value (counts below 200) resulted from the inclusion of *Daphnia truncata*, which is a plankter from high UV saline habitats in Australia. Unlike the finding with photolesion promoters, trinucleotide “TT” containing motifs associated with a lack or inhibition of photolesions^31^ (TTC/TTG) showed slightly greater (*Scapholeberis*) or largely overlapping counts (*Megafenestra*) to those of planktonic daphniids and moinids.

## Discussion

Our extensive geographic sampling and genetic evidence suggests that despite a simple monolayered habitat, the biogeography of the neustonic Scapholeberinae is complex involving several regional clades and species. Two species groups, *S. mucronata* and *S. rammneri*, include most of the taxa and most of the populations of the genus. Species that appear to have multicontinental distributions such as *S. rammneri*, *S. mucronata*, and *S. kingii* are indeed an amalgam of regional clades. *S. kingii* may indeed be a species “group” (as with *S. rammneri*, *S. mucronata)* with more than the two allopatric clades. African “*S. kingii*”, which we did not sample, is a candidate for such an additional allopatric clade.

The detection of several closely related regional clades on both sides of the former Bering land bridge could suggest recent (ca. Late Pleistocene) connectivity. The finding of these novel Beringian lineages also highlights the importance of Beringian ponds to freshwater diversity at a time when the region’s freshwater ecosystems are threatened by global changes (such as permafrost thawing). Beringia has been proposed as an important Pleistocene refugium, dispersion center, and dispersion corridor for freshwater animals^34–36^. The lack of a subarctic Beringian clade in the multicontinental *S. rammneri* may be due to its apparent temperate habitat preferences. Indeed, *S. rammneri* appears to have a temperate range limit in the northern and the southern hemispheres (Fig. 1). The pattern is unlikely to be a sampling artifact as we have carried out extensive multiyear sampling in northwest Alaska, Chukotka, and Kamchatka without detecting *S. rammneri*.

Our results are the first indication that *S. microcephala* occurs in North America (Alaska), that *S. rammneri* is widespread in South America, and that a lineage related to *S. freyi* occurs in Europe. Newly discovered disjunct populations are often suspected to be recent anthropogenic introductions in the Cladocera. Indeed, such introductions have been frequent in both North America and South America (e.g., *D. pulex* and *D. lumholtzi*). However, the evidence for recent anthropogenic introductions in the present cases is lacking.

*S. microcephala* is a rare species that has been detected previously from dystrophic waters (such as in bogs) of the western Palearctic^6^. We also detected this species in dystrophic waters (tundra thaw ponds) near the tree line in northwest Alaska. The apparent absence of *S. microcephala* in the central and the eastern Palearctic could be a sampling artifact. Also, a direct comparison of Alaskan and Western Palearctic specimens is needed to rule out the possibility that the Alaskan populations represent a cryptic endemic. We have used “*S. cf. microcephala”* for the Alaskan populations to reflect this uncertainty in taxonomic assignment. The monophyly of the broadly distributed South American *S. rammneri* and their strongly supported genetic nesting within western populations of North American *S. rammneri* is consistent with recent connectivity in the direction of western North America to South America. An analysis with nuclear genes would be informative to further test the timing of introduction and geographic source of South American *S. rammneri*. Our findings also suggest that reports of *S. kingii* from South America may have been confused with *S. rammneri*.

Recently introduced populations of cladocerans rarely show the haplotype diversity that we observe here^18,23,27^. Introduced *Bosmina coregoni*, *Daphnia pulex* (Panarctic) and *Daphnia lumholtzi*, for example, shared mitochondrial haplotypes across the continents (including North America to South America introductions). We used a conserved section of the mitochondrial genome for *S. rammneri* but still found considerable endemic genetic structure in the South American populations. The nested phylogenetic position of a monophyletic South American *S. rammneri* clade within the western North America grouping is consistent with colonization from North America to South America. However, the divergent genetic structure within the South American samples appears inconsistent with post-European contact introductions.

Finally, the detection of the New World “*freyi*”-like lineage in Europe is unexpected. As the European haplotype is basal to the closely related specimens from Eastern North America, there is no evidence of an introduction from North America to Europe. The similarity to *S. freyi* extends beyond the rDNA gene regions as a protein coding gene (COI) showed 92% similarity to *S. freyi* (from Brazil). As such, the European sample could have resulted from an introduction from a lineage that we failed to detect in the New World.

The robust structure in the introduced South American *S. rammneri* could be due to an unusually rapid rate of molecular evolution within *Scapholeberis*. Indeed, our evidence supports an ongoing rapid rate of evolution for the mitochondrial genes that we examined. Our tests indicated that there is little evidence that the relatively rapid evolutionary rate in *Scapholeberis* is due to GC-content shifts, alignment artifacts (based on a lack of base composition heterogeneity and the removal of less than 11% of sites by the GBLOCKs alignment noise filter), pseudogenes, or overparameterized models. Nevertheless, the relative genetic divergence within *Scapholeberis* is as great as among families of cladocerans and more than twice the divergence found in other daphniid genera. Morphologically cryptic species that have the mitochondrial divergence at the family level for cladocerans is very unusual. Pervasive strong divergences within the genus *Scapholeberis* suggest that the rate acceleration goes beyond a single branch. Rapid rates of evolution could lead to a systematic bias that misleads phylogeny and biogeographic conclusions about the timing of introductions. In such a case, sampling more genes could reinforce the bias and potentially provide strong support for an erroneous grouping. More taxonomic sampling for the nuclear genes that show support for a basal *Scapholeberis* position needs to be carried out to determine if a widespread rate acceleration bias also affects nuclear genes. As such, it seems premature to conclude that neustonic daphniids evolved more than once. The lack of a subfossil and a fossil record (or other sources of calibration) for the Scapholeberinae remains a limitation for estimating rates directly.

The existence of ancient mitochondrial pseudogenes can create the appearance of accelerated lineages. However, it is unlikely that nuclear pseudogenes explain the observed rate acceleration in the present study for several reasons. First, the rate acceleration appears to affect most or all of the monophyletic lineages in *Scapholeberis*. It is unlikely that nuclear copies would markedly exceed the rates of evolution in daphniid mitochondrial DNA. Also, there is only one significant match (likely mitochondrial) in genomic contigs of *Scapholeberis* to sequences from the present study – and this is a full-length match with only five substitutions. The presumptive mitochondrial contig also contains a protein-coding gene, ND2, which has an open reading frame that is inconsistent with the existence of an ancient pseudogene.

The markedly lower numbers of salient photolesion-promoting trinucleotides in neustonic versus planktonic daphniids and moinids is consistent with the UVR adapted genome hypothesis. However, it is currently unknown if these differences affect the formation of photolesions in neustonic daphniids or if the pattern applies to less AT-rich regions (i.e., the nuclear genome). Notably, the sole planktonic daphniid with low TTA/TTT counts (below 200) was also the sole zooplankter in our data from a high energy habitat, *Daphnia truncata*^33^. If *Megafenestra* and *Scapholeberis* independently evolved the neustonic habit then there are three UVR habitat contrasts in our data that show pronounced reductions in salient trinucleotides.

While many of the new-found lineages in the present study are assignable to valid species (based on morphology and geography), there are several divergent lineages where the taxonomy remains unclear. For example, individuals of the Beringian clade of *Megafenestra* appear to have some diagnostic morphologies of both *M. nasuta* and *M. aurita*. Most probably, this Beringian taxon was reported as *M*. cf. *nasuta* from Central Yakutia^37^. The taxonomy of the Eastern Palearctic clade related to *S. kingii* would also benefit from a detailed study of all life stages and morphology – no diagnostic morphological differences for this taxon were detected in parthenogenetic females^38^. Males are potentially more valuable for taxonomy, but they are not described in the Far Eastern *S*. cf. *kingii*. The eastern Nearctic *Scapholeberis* cf. *armata* appears to have the diagnostic rostrum and mucro spine traits of *S. armata*^6^. However, the type location of this species is in Minnesota and the type specimens are lost. As we used only mitochondrial markers, there has been no assessment of the proposed hybridization within the *S. armata* complex^6^. Because it remains challenging to distinguish divergent genetic lineages of *Scapholeberis* based on morphology, it would seem that increases in the rates of rRNA gene evolution are not associated with increased morphological evolution. Still, there is much work to be done to revise the taxonomy of the Scapholeberinae using combined morphological and genetic markers.

As with other cladocerans, the biology of geographic range limits remains enigmatic in the Scapholeberinae. Why are there sharp geographic boundaries for some clades of *S. rammneri* and *S. mucronata* when passive dispersal appears to have resulted in postglacial range expansion over thousands of kilometers? When it is considered that *S. rammneri* has likely colonized South America from western North America and spread to occupy a substantial Neotropical range, the dispersal limitation explanations are weakened. The existence of strong priority effects and expansion from few glacial refugia may explain some of this abutting geographic clade pattern for widespread species.

## Conclusions

Neustonic daphniids have a complex biogeography with more geographic lineages than previously appreciated. We detected at least six intercontinental lineages with four being Transberingian. Thus, Beringia, with its rapidly changing permafrost ponds contains a hidden diversity of freshwater neustonic daphniids. We found evidence for an introduction from western North America to Patagonia in the *S. rammneri* group. Surprisingly, the pronounced population structure in Patagonia appears inconsistent with a recent anthropogenic introduction. The neustonic daphniids failed to form a monophyletic group, but the rates of mitochondrial rRNA genes in the genus *Scapholeberis* appear to be pervasively accelerated compared to other daphniids. Biogeographic and phylogenetic studies of neustonic daphniids using these genes should consider an increased evolutionary rate.

## Methods

### Sample collection

Samples were collected using a small throw net or D-net. 402 new sequences were obtained from 186 global populations of daphniids with the emphasis on the neustonic Scapholeberinae (Table S1; Fig. 1a–d). Species were identified, sorted, and immediately frozen in the field using a dry shipper or placed in 100% ethanol. Samples were stored at −80 °C until the time of DNA extraction. We used the existing taxonomic keys for the Scapholeberinae^6^ but identified *Scaphleberis freyi* as a species instead of a subspecies of *S. armata*. We used the term “cf.” for geographic clades that have uncertain morphological divergence from named species.

### PCR and sequencing techniques

DNA of single individuals was extracted using DNA QuickExtract (Epicenter). The individual was ground in the extraction medium and incubated for 1 hr at 60 °C and 7 min at 94 °C on a Stratagene RoboCycler (Stratagene, Inc., La Jolla, CA). The sample was stored at −20 °C until the time of DNA amplification. Each polymerase chain reaction (PCR) for DNA amplification was performed in a total volume of 50 μl containing 36.5 μl irradiated H_2_O, 5 μl 10X buffer, 1 μl dNTP’s, 0.75 μl of each primer, 1 μl *Taq* DNA polymerase, and 5 μl of DNA template (~200 ng μl^−1^). Primers used to amplify the mitochondrial 12S rRNA-16S rRNA gene target fragments were: 12S 5′-AAAACCAGGATTAGATACCCTGTTAT-3′ and 16S 5′-CCTCACTAAAGGGATGATTATGCTACCTTAGCACAGTC-3′. These primers and two additional primers (12SINT 5′-AACCCTGATACACAAGGTACGA-3′ and 16SINT 5′-CAAAGTAGATGTACTGGAA-3′) were used for Sanger sequencing.

The PCR conditions for DNA amplification were set to 1 cycle: 2 min at 94 °C; 40 cycles: 45 sec at 94 °C, 45 sec at 50 °C, 75 sec at 72 °C; and 1 cycle: 8 min at 72 °C on a MJ Research MiniCycler (MJ Research, Inc., Boston, MA). PCR products were gel purified and sequenced in both directions, totaling approximately 1200 base pairs across the 12S-16S region (including the tRNA^val^ region). Sequence data from this study are available from GenBank MK582616–MK583010 and MN066622–MN066634.

DNA sequences were assembled and edited using Geneious 7.1 (https://www.geneious.com). The alignment was carried out in the online version of MAFFT 7^39^ using the default form. Haplotype networks were estimated and visualized using the median joining algorithm implementation of PopART 1.7^40^. Non-stationarity in base composition was assessed with a base composition heterogeneity chi-squared test in IQtree 1.6. Substitution models were chosen by the ModelFinder routine with the Bayesian Information Criterion (BIC) in IQtree 1.6^41^. The effects of heterotachy (differing rates of evolution among lineages) on phylogeny were assessed using the General Heterogeneous evolution On a Single Topology (GHOST) model of sequence evolution (GTR + FO*H4 in IQtree^41^). Phylogenetic trees were estimated using the Maximum Likelihood algorithm in IQtree and Seaview 4.7^42^. Support values estimated were aLRT, nonparametric bootstraps, and the transfer bootstrap expectation method (using BOOSTER: https://booster.pasteur.fr/)^43^. Genomic sequences were compared to published mitochondrial genomes to verify mitochondrial origins. Haplotype diversity and the average number of genetic differences among clades were calculated using DNAsp 6^44^. The non-anomopod cladoceran genome of *Diaphanosoma dubium* was used for outgroup rooting. The Relative Timetree procedure was carried out using MEGA X^45^ with sequences of *Scapholeberis*, *Megafenestra*, and other daphniids (representing all known genera and subgenera) and moinids. For comparison of UVR photolesion salient trinucleotides, three datasets were created: *Scapholeberis*, *Megafenestra*, and planktonic daphniids/moinids (most of these were sequenced for this study; see the Genbank Accessions numbers below and in Supplementary Data). Each alignment was then exposed to the “dealign” option in Seaview to remove internal gaps. Trinucleotide counts (TTA,TTT,TTC,TTG) were summed for each sequence for the plus and minus strands (i.e., complements to the salient motifs). A simple Python script was used to count and sum motifs. Note that every instance of a motif is counted such that the sequence “TTTT” contains two “TTT” trinucleotide motifs. Box and whiskers plots were made in R comparing the three groups for counts of photolesion promoting motifs (TTA/TTT) and inhibiting motifs (TTC/TTG).

## Electronic supplementary material

Supplementary Informaton.

## Data Availability

Voucher specimens have been deposited into AAK’s private collection. DNA sequences were submitted to Genbank with the following accession numbers (MK582616–MK583010 and MN066622–MN066634).
