# Supplementary material for: The Intercontinental phylogeography of neustonic daphniids
Source: Sci Rep. 2020 Feb 4;10:1818. doi: 10.1038/s41598-020-58743-8 (PMC7000678; doi:10.1038/s41598-020-58743-8)
Supplement: Supplementary file 1 — Supplementary Informaton. [file 41598_2020_58743_MOESM1_ESM.pdf]

Supplementary Information for:

The Intercontinental phylogeography of neustonic daphniids

*Derek J. Taylor[1], Sandra J. Connelly[2], and Alexey A. Kotov[3]

[1] Department of Biological Sciences, The State University of New York at Buffalo, Buffalo, NY 14260, USA.

[2] Thomas H. Gosnell School of Life Sciences, Rochester Institute of Technology, Rochester, NY, USA.

[3] A. N. Severtsov Institute of Ecology and Evolution, Leninsky Prospect 33, 119071, Moscow, Russia.

***Correspondence:** Derek J. Taylor, djtaylor@buffalo.edu

Table S1. Details for the specimens of daphniid crustaceans sequenced in the present study. Accession numbers are provided here (for previously published sequences) and in the data availability section for all other sequences (i.e., from this study).

Clade	Taxon	Sequence ID	Country	State	Locality	Collector	N	E
A	mucronata1	Poland_1a	Poland		Jegocin Lake	D.J. Taylor, M. Faustova, A. Petrusek, V. Sacherova	53.6651	21.7034
A	mucronata1	Germany_1a	Germany	Branderburg	Sckamützelsee	M Belyaeva	52.2333	14.05
A	mucronata1	Czechia_2a	Czech Republic		Fish pond Zoldanka, W of Blatna	V. Kořínek	49.42	13.82
A	mucronata1	Czechia_2a	Czech Republic		Fish pond Zoldanka, W of Blatna	V. Korinek	49.42	13.82
A	mucronata1	Czechia_3a	Czech Republic		A forest pond in a clamp of alders, W of Blatna, NE of Cecelovice, Strakonice District	V. Korinek	49.42	13.82
A	mucronata1	Germany_1b	Germany	Branderburg	Sckamützelsee	M Belyaeva	52.2333	14.05
A	mucronata1	Germany_1c	Germany	Branderburg	Sckamützelsee	M Belyaeva	52.2333	14.05
A	mucronata1	Czechia_1a	Czech Republic		Pond in Pazderna	A. Petrusek	49.7166	18.4569
A	mucronata1	Czechia_1b	Czech Republic		Pond in Pazderna	A. Petrusek	49.7166	18.4569
A	mucronata1	Belgium_2a	Belgium		Lake Donk		51.03	3.98
A	mucronata1	Belgium_2b	Belgium		Lake Donk		51.03	3.98
A	mucronata1	Belgium_1a	Belgium		Nieuw Donk, Overmere-Berlare	K. Van Damme	51.03703	3.981533
A	mucronata1	Belgium_1b	Belgium		Nieuw Donk, Overmere-Berlare	K. Van Damme	51.03703	3.981533
A	mucronata1	France_1a	France		Pouan-Les-Vallée, temporary pond	J. F. Cart	48.54361	4.056944
A	mucronata1	Belgium_1c	Belgium		Nieuw Donk, Overmere-Berlare	K. Van Damme	51.03703	3.981533
A	mucronata1	Belgium_1d	Belgium		Nieuw Donk, Overmere-Berlare	K. Van Damme	51.03703	3.981533
A	mucronata1	Belgium_1e	Belgium		Nieuw Donk, Overmere-Berlare	K. Van Damme	51.03703	3.981533
A	mucronata1	Belgium_1f	Belgium		Nieuw Donk, Overmere-Berlare	K. Van Damme	51.03703	3.981533
A	mucronata1	Russia_Kaliningrad_1a	Russia (European)	Kaliningrad Area	A pond, Curonian Spit	V. Smirnov, O. Smirnova & V. Smirnova	55.15738	20.84447
A	mucronata1	France_2a	France		Marnay, Le grand Mort	J. F. Cart	48.51722	3.556389
A	mucronata1	France_2b	France		Marnay, Le grand Mort	J. F. Cart	48.51722	3.556389
A	mucronata1	Russia_Kaliningrad_1b	Russia (European)	Kaliningrad Area	A pond, Curonian Spit	V. Smirnov, O. Smirnova & V. Smirnova	55.15738	20.84447
A	mucronata1	Russia_Kaliningrad_1c	Russia (European)	Kaliningrad Area	A pond, Curonian Spit	V. Smirnov, O. Smirnova & V. Smirnova	55.15738	20.84447
A	mucronata1	Belgium_1g	Belgium		Nieuw Donk, Overmere-Berlare	K. Van Damme	51.03703	3.981533
A	mucronata1	France_2c	France		Marnay, Le grand Mort	J. F. Cart	48.51722	3.556389
A	mucronata1	France_2d	France		Marnay, Le grand Mort	J. F. Cart	48.51722	3.556389
B	mucronata3	USA_AK_10a	U.S.A.	Alaska	Pond 4a near Teller		65.2418	-166.3296
B	mucronata3	USA_AK_12a	U.S.A.	Alaska	Pond 1 near Teller		n/a	n/a
B	mucronata3	Russia_Tomsk_1a	Russia (Asian)	Tomsk Area	Lake Boyarskoe, town of Tomsk	AA Kotov	56.4536	84.9135
B	mucronata3	Russia_Khakassia_1f	Russia (Asian)	Khakass Autonomous Republic	Oxbow near Izykhskie Kopi, Abakan River	D. E. Shcherbakov	53.55582	91.27282
B	mucronata3	USA_AK_9a	U.S.A.	Alaska	Pond 4 near Teller	D. J. Taylor, A. A. Kotov, M. Ballinger & A. Medeiros	65.17032	-166.3022
B	mucronata3	Mongolia_2a	Mongolia	Olgiy Aimag	A affluen of unnamed small river, which is an affluent of Hoton-Nuur Lake, Mongolian Altai	D.P. Karabanov	48.548	88.46861
B	mucronata3	Mongolia_2b	Mongolia	Olgiy Aimag	A affluen of unnamed small river, which is an affluent of Hoton-Nuur Lake, Mongolian Altai	D.P. Karabanov	48.548	88.46861
B	mucronata3	Russia_Murmansk_1a	Russia (European)	Murmansk Area	наскальная лужа, о. Кастьян, окрестности ББС МГУ	E. I. Bekker	66.49	33.38
B	mucronata3	Russia_Karelia_1b	Russia (European)	Karelia Autonomous Republic	Keret' Lake System	A. N. Reshetnikov	65.79361	32.69111
B	mucronata3	Russia_Karelia_1a	Russia (European)	Karelia Autonomous Republic	Keret' Lake System	A. N. Reshetnikov	65.79361	32.69111
B	mucronata3	Russia_Tomsk_1b	Russia (Asian)	Tomsk Area	Lake Boyarskoe, town of Tomsk	AA Kotov	56.4536	84.9135
B	mucronata3	Russia_Tomsk_2a	Russia (Asian)	Tomsk Area	Petrovskaja Protoka	AA Kotov	56.5878	84.8374
B	mucronata3	Russia_Tomsk_3a	Russia (Asian)	Tomsk Area	Lake Kurok	AA Kotov	56.8142	84.5606
B	mucronata3	Russia_Tomsk_3b	Russia (Asian)	Tomsk Area	Lake Kurok	AA Kotov	56.8142	84.5606
B	mucronata3	Russia_Tomsk_3c	Russia (Asian)	Tomsk Area	Lake Kurok	AA Kotov	56.8142	84.5606
B	mucronata3	Russia_Tomsk_3d	Russia (Asian)	Tomsk Area	Lake Kurok	AA Kotov	56.8142	84.5606
B	mucronata3	Russia_Pskov_1a	Russia (European)	Pskov Area	Lake Glukhoe near village of Zhizhitsa, Zhizhitskaya Volost'	O. A. Krylovich	56.28653	31.35989
B	mucronata3	Russia_Pskov_1b	Russia (European)	Pskov Area	Lake Glukhoe near village of Zhizhitsa, Zhizhitskaya Volost'	O. A. Krylovich	56.28653	31.35989
B	mucronata3	Russia_Tver_1a	Russia (European)	Tver Area	Leke Pesno, region of Zapadnaya Dvina town	A. A. Kotov & Y. R. Galimov	56.32553	31.9178
B	mucronata3	Russia_Chelyabinsk_1a	Russia (European)	Chelyabinsk Area	A swamp near Katav-Ivanovsk Pond	A. A. Kotov	54.75063	58.22517
B	mucronata3	Russia_Tomsk_5a	Russia (Asian)	Tomsk Area	A small swamp, righ side of road Kolpashevo-Parabel'	AA Kotov	58.1353	82.6264
B	mucronata3	Russia_Tomsk_5b	Russia (Asian)	Tomsk Area	A small swamp, righ side of road Kolpashevo-Parabel'	AA Kotov	58.1353	82.6264
B	mucronata3	Russia_Chelyabinsk_2a	Russia (European)	Chelyabinsk Area	Porogi Water Reservoir, on River Bol'shaja Satka (affluent of the Ai River)	A. A. Kotov	55.27532	59.13388
B	mucronata3	Russia_YamaloNenets_1a	Russia (Asian)	Yamalo-Nenets Autonomour Area	Un-named lake 1 in Nadymsky Gorodok, 31 km from Obskaya Guba	A. B. Savinetsky	66.0602	72.00614
B	mucronata3	Russia_YamaloNenets_2a	Russia (Asian)	Yamalo-Nenets Autonomour Area	Un-named lake 1 in Nadymsky Gorodok, 31 km from Obskaya Guba	A. B. Savinetsky	66.0602	72.00614
B	mucronata3	Russia_Tomsk_5c	Russia (Asian)	Tomsk Area	A small swamp, righ side of road Kolpashevo-Parabel'	AA Kotov	58.1353	82.6264
B	mucronata3	Russia_Tomsk_6a	Russia (Asian)	Tomsk Area	Sukhozhinskoe Lake, Parabel'	AA Kotov	58.6849	81.5157

B	mucronata3	Russia_Pskov_2a	Russia (European)	Pskov Area	A small pond 50 m from shore of Chudskoe Lake, near village of Zapol'je	A. A. Kotov & Y. R. Galimov	58.63642	27.78997
B	mucronata3	Russia_Pskov_3a	Russia (European)	Pskov Area	Lake Sinovets near village of Panovo	A. A. Kotov & Y. R. Galimov	56.59742	29.01383
B	mucronata3	Russia_Tomsk_6b	Russia (Asian)	Tomsk Area	Sukhozhinskoe Lake, Parabel'	AA Kotov	58.6849	81.5157
B	mucronata3	Russia_Tomsk_6c	Russia (Asian)	Tomsk Area	Sukhozhinskoe Lake, Parabel'	AA Kotov	58.6849	81.5157
B	mucronata3	Russia_Tomsk_6d	Russia (Asian)	Tomsk Area	Sukhozhinskoe Lake, Parabel'	AA Kotov	58.6849	81.5157
B	mucronata3	Russia_Saratov_1a	Russia (European)	Saratov Area	A bay of River Eruslan near village of Diakovka	L. E. Savinetskaya	50.7355	46.7797
B	mucronata3	Russia_Saratov_1b	Russia (European)	Saratov Area	A bay of River Eruslan near village of Diakovka	L. E. Savinetskaya	50.7355	46.7797
B	mucronata3	Russia_Saratov_1c	Russia (European)	Saratov Area	A bay of River Eruslan near village of Diakovka	L. E. Savinetskaya	50.7355	46.7797
B	mucronata3	Russia_Saratov_1d	Russia (European)	Saratov Area	A bay of River Eruslan near village of Diakovka	L. E. Savinetskaya	50.7355	46.7797
B	mucronata3	Russia_Saratov_1e	Russia (European)	Saratov Area	A bay of River Eruslan near village of Diakovka	L. E. Savinetskaya	50.7355	46.7797
B	mucronata3	Russia_Tomsk_7a	Russia (Asian)	Tomsk Area	A puddle near River Ob', Stariy Vartovsk	AA Kotov	60.8366	76.6805
B	mucronata3	Russia_Tomsk_7b	Russia (Asian)	Tomsk Area	A puddle near River Ob', Stariy Vartovsk	AA Kotov	60.8366	76.6805
B	mucronata3	Russia_Tomsk_7c	Russia (Asian)	Tomsk Area	A puddle near River Ob', Stariy Vartovsk	AA Kotov	60.8366	76.6805
B	mucronata3	Russia_Tomsk_7d	Russia (Asian)	Tomsk Area	A puddle near River Ob', Stariy Vartovsk	AA Kotov	60.8366	76.6805
B	mucronata3	Russia_Omsk_1a	Russia (Asian)	Omsk Area	Tatyn, an old-bed affluent of River Ishim	A. A. Kotov	57.64382	71.16827
B	mucronata3	Russia_Tomsk_3e	Russia (Asian)	Tomsk Area	Lake Kurok	AA Kotov	56.8142	84.5606
B	mucronata3	Russia_Chelyabinsk_3a	Russia (Asian)	Chelyabinsk Area	Lake Pestchanoe, the Tobol Basin	A. A. Kotov	54.99102	59.83963
B	mucronata3	Russia_Sarstov_2a	Russia (European)	Saratov Area	A pool in Diakovka willage	L. E. Savinetskaya and Co	50.71032	46.76908
B	mucronata3	Russia_Sarstov_2b	Russia (European)	Saratov Area	A pool in Diakovka willage	L. E. Savinetskaya and Co	50.71032	46.76908
B	mucronata3	Russia_Penza_1a	Russia (European)	Penza Area	A pond near Armijovo	E. I. Bekker	52.88081	45.48056
B	mucronata3	Russia_Penza_1b	Russia (European)	Penza Area	A pond near Armijovo	E. I. Bekker	52.88081	45.48056
B	mucronata3	Russia_Penza_2a	Russia (European)	Penza Area	A lake fully covered by vegetation, locality Montazhny, viciniyies of Penza	E. I. Bekker	53.218	45.125
B	mucronata3	Russia_Karelia_2a	Russia (European)	Karelia Autonomous Republic	Rockpool near Ladoga Lake	Я. Р. Галимов	61.47071	30.42926
B	mucronata3	Russia_Penza_2b	Russia (European)	Penza Area	A lake fully covered by vegetation, locality Montazhny, viciniyies of Penza	E. I. Bekker	53.218	45.125
B	mucronata3	Russia_Khakassia_1b	Russia (Asian)	Khakass Autonomous Republic	Oxbow near Izykhskie Kopi, Abakan River	D. E. Shcherbakov	53.55582	91.27282
B	mucronata3	Russia_Khakassia_1c	Russia (Asian)	Khakass Autonomous Republic	Oxbow near Izykhskie Kopi, Abakan River	D. E. Shcherbakov	53.55582	91.27282
B	mucronata3	Russia_Tver_3a	Russia (European)	Tver Area	A metal tank, Kuznetsovo village	T.P. Korobkova	57.31	36.1
B	mucronata3	Russia_Penza_2c	Russia (European)	Penza Area	A lake fully covered by vegetation, locality Montazhny, viciniyies of Penza	E. I. Bekker	53.218	45.125
B	mucronata3	Russia_Bryansk_1a	Russia (European)	Bryansk Area	A small lake, Kokino, Vygonichsky District	E. Y. Blagoveschenskaya	53.15033	34.11189
B	mucronata3	Russia_Karelia_2b	Russia (European)	Karelia Autonomous Republic	Rockpool near Ladoga Lake	Я. Р. Галимов	61.47071	30.42926
B	mucronata3	Russia_Karelia_2c	Russia (European)	Karelia Autonomous Republic	Rockpool near Ladoga Lake	Я. Р. Галимов	61.47071	30.42926
B	mucronata3	Russia_Penza_2d	Russia (European)	Penza Area	A lake fully covered by vegetation, locality Montazhny, viciniyies of Penza	E. I. Bekker	53.218	45.125
B	mucronata3	Russia_Penza_2e	Russia (European)	Penza Area	A lake fully covered by vegetation, locality Montazhny, viciniyies of Penza	E. I. Bekker	53.218	45.125
B	mucronata3	Mongolia_4d	Mongolia	Selenge Aimag	Affluent of the Orkhon River	D. P. Karabanov	50.24805	106.1406
B	mucronata3	Russia_Arkhangelsk_1a	Russia (European)	Arkhangelsk Area	Lake near Kumichevo Kordon, Pinega Natural Reservoir	E. I. Bekker	64.57514	42.9629
B	mucronata3	Russia_NizhnyNovgorod_1a	Russia (European)	Nizhni Novgorod Area	Sandy mine lake, Dalnekonstantinovsk District	A. B. Savinetsky	55.75912	43.72533
B	mucronata3	Russia_Tver_3b	Russia (European)	Tver Area	A metal tank, Kuznetsovo village	T.P. Korobkova	57.31	36.1
B	mucronata3	Russia_Tver_3c	Russia (European)	Tver Area	A metal tank, Kuznetsovo village	T.P. Korobkova	57.31	36.1
B	mucronata3	Russia_Tver_3d	Russia (European)	Tver Area	A metal tank, Kuznetsovo village	T.P. Korobkova	57.31	36.1
B	mucronata3	Russia_Bryansk_1b	Russia (European)	Bryansk Area	A small lake, Kokino, Vygonichsky District	E. Y. Blagoveschenskaya	53.15033	34.11189
B	mucronata3	Russia_Bryansk_1c	Russia (European)	Bryansk Area	A small lake, Kokino, Vygonichsky District	E. Y. Blagoveschenskaya	53.15033	34.11189
B	mucronata3	Russia_Bryansk_1d	Russia (European)	Bryansk Area	A small lake, Kokino, Vygonichsky District	E. Y. Blagoveschenskaya	53.15033	34.11189
B	mucronata3	Russia_NizhnyNovgorod_1b	Russia (European)	Nizhni Novgorod Area	Sandy mine lake, Dalnekonstantinovsk District	A. B. Savinetsky	55.75912	43.72533
B	mucronata3	Russia_Tver_3e	Russia (European)	Tver Area	A metal tank, Kuznetsovo village	T.P. Korobkova	57.31	36.1
B	mucronata3	Mongolia_4b	Mongolia	Selenge Aimag	Affluent of the Orkhon River	D. P. Karabanov	50.24805	106.1406
B	mucronata3	Russia_Saratov_1f	Russia (European)	Saratov Area	A bay of River Eruslan near village of Diakovka	L. E. Savinetskaya	50.7355	46.7797
B	mucronata3	Russia_Penza_1c	Russia (European)	Penza Area	A pond near Armijovo	E. I. Bekker	52.88081	45.48056
B	mucronata3	Russia_Tver_3f	Russia (European)	Tver Area	A metal tank, Kuznetsovo village	T.P. Korobkova	57.31	36.1

B	mucronata3	Russia_Yakutia_3a	Russia (Asian)	Yakutia Autonomous Republic	A channel 1 near Kuria Strelka, town of Yakutsk	А. А. Котов, А. И. Климовский	62.01894	129.7567
B	mucronata3	Russia_Khakassia_1d	Russia (Asian)	Khakass Autonomous Republic	Oxbow near Izykhskie Kopi, Abakan River	D. E. Shcherbakov	53.55582	91.27282
B	mucronata3	Russia_Khakassia_1e	Russia (Asian)	Khakass Autonomous Republic	Oxbow near Izykhskie Kopi, Abakan River	D. E. Shcherbakov	53.55582	91.27282
B	mucronata3	Russia_Khakassia_1g	Russia (Asian)	Khakass Autonomous Republic	Oxbow near Izykhskie Kopi, Abakan River	D. E. Shcherbakov	53.55582	91.27282
B	mucronata3	Russia_Karelia_2d	Russia (European)	Karelia Autonomous Republic	Rockpool near Ladoga Lake	Я. Р. Галимов	61.47071	30.42926
B	mucronata3	Russia_Sarstov_2c	Russia (European)	Saratov Area	A pool in Diakovka willage	L. E. Savinetskaya and Co	50.71032	46.76908
B	mucronata3	Russia_Khakassia_1h	Russia (Asian)	Khakass Autonomous Republic	Oxbow near Izykhskie Kopi, Abakan River	D. E. Shcherbakov	53.55582	91.27282
B	mucronata3	Russia_Tver_2a	Russia (European)	Tver Area	Pukhlinskiy Stvor, Uglich Water Reservoir	Ivanovsky	57.0163	37.4428
B	mucronata3	Russia_Komi_1a	Russia (European)	Komi Autonomous Republic	Lake Yelya-ty, River Sysola basin	O. N. Kononova	61.58333	50.78333
B	mucronata3	Russia_Evenki_1a	Russia (Asian)	Evenki Autonomous Area	An almost dried puddle in the Nizhniaya Tunguska walley	D. E. Shcherbakov	64.14072	101.7657
B	mucronata3	Russia_Evenki_1b	Russia (Asian)	Evenki Autonomous Area	An almost dried puddle in the Nizhniaya Tunguska walley	D. E. Shcherbakov	64.14072	101.7657
B	mucronata3	Russia_Karelia_2e	Russia (European)	Karelia Autonomous Republic	Rockpool near Ladoga Lake	Я. Р. Галимов	61.47071	30.42926
B	mucronata3	Russia_Tver_2b	Russia (European)	Tver Area	Pukhlinskiy Stvor, Uglich Water Reservoir	Ivanovsky	57.0163	37.4428
B	mucronata3	Russia_Komi_1b	Russia (European)	Komi Autonomous Republic	Lake Yelya-ty, River Sysola basin	O. N. Kononova	61.58333	50.78333
B	mucronata3	Russia_Yakutia_7b	Russia (Asian)	Yakutia Autonomous Republic	Lake 1 on Melnikova Island, the Lena River	A. A. Kotov	63.85302	127.4713
B	mucronata3	Russia_Yakutia_7c	Russia (Asian)	Yakutia Autonomous Republic	Lake 1 on Melnikova Island, the Lena River	A. A. Kotov	63.85302	127.4713
B	mucronata3	Russia_Yakutia_8a	Russia (Asian)	Yakutia Autonomous Republic	Lake 3 (Kvadratnoe) on Mel'nikov Island, the Lena River	A. A. Kotov	63.85395	127.4678
B	mucronata3	USA_AK_9b	U.S.A.	Alaska	Pond 4 near Teller	D. J. Taylor, A. A. Kotov, M. Ballinger & A. Medeiros	65.17032	-166.3022
B	mucronata3	Russia_Yakutia_8b	Russia (Asian)	Yakutia Autonomous Republic	Lake 3 (Kvadratnoe) on Mel'nikov Island, the Lena River	A. A. Kotov	63.85395	127.4678
B	mucronata3	Russia_Yakutia_8c	Russia (Asian)	Yakutia Autonomous Republic	Lake 3 (Kvadratnoe) on Mel'nikov Island, the Lena River	A. A. Kotov	63.85395	127.4678
B	mucronata3	Russia_YamaloNenets_3a	Russia (Asian)	Yamalo-Nenets Autonomour Area	Lake 2 in Nadymsky Gorodok, 31 km from Obskaya Guba	A. B. Savinetsky	66.2208	72.04506
B	mucronata3	Mongolia_3a	Mongolia	Bayankhongor Aimag	Affluent of the Tuin-Gol River near town of Bayankhongor	D. P. Karabanov	46.30889	100.7464
B	mucronata3	Russia_Yakutia_9a	Russia (Asian)	Yakutia Autonomous Republic	Vtoroe Ozero (Abycha) near Sangar	A.A. Kotov	63.94306	127.5128
B	mucronata3	Russia_Yakutia_9b	Russia (Asian)	Yakutia Autonomous Republic	Vtoroe Ozero (Abycha) near Sangar	A.A. Kotov	63.94306	127.5128
B	mucronata3	Russia_Yakutia_9c	Russia (Asian)	Yakutia Autonomous Republic	Vtoroe Ozero (Abycha) near Sangar	A.A. Kotov	63.94306	127.5128
B	mucronata3	USA_AK_9c	U.S.A.	Alaska	Pond 4 near Teller	D. J. Taylor, A. A. Kotov, M. Ballinger & A. Medeiros	65.17032	-166.3022
B	mucronata3	Russia_Tver_1b	Russia (European)	Tver Area	Leke Pesno, region of Zapadnaya Dvina town	A. A. Kotov & Y. R. Galimov	56.32553	31.9178
B	mucronata3	Russia_YamaloNenets_4a	Russia (Asian)	Yamalo-Nenets Autonomour Area	A littoral of lake at Mestorozdeniye Russkoye	P. M. Glazov	66.64943	80.55222
C	mucronata2	USA_AK_11a	U.S.A.	Alaska	Pond 17 near Teller		n/a	n/a
C	mucronata2	USA_AK_13a	U.S.A.	Alaska	Pond 14 near Taylor	D. J. Taylor, A. A. Kotov, M. Ballinger & A. Medeiros	65.36631	-164.6751
C	mucronata2	USA_AK_13b	U.S.A.	Alaska	Pond 14 near Taylor	D. J. Taylor, A. A. Kotov, M. Ballinger & A. Medeiros	65.36631	-164.6751
C	mucronata2	USA_AK_13c	U.S.A.	Alaska	Pond 14 near Taylor	D. J. Taylor, A. A. Kotov, M. Ballinger & A. Medeiros	65.36631	-164.6751
C	mucronata2	USA_AK_14a	U.S.A.	Alaska	Pond 10 near Taylor	D. J. Taylor, A. A. Kotov, M. Ballinger & A. Medeiros	65.39655	-164.6606
C	mucronata2	USA_AK_14b	U.S.A.	Alaska	Pond 10 near Taylor	D. J. Taylor, A. A. Kotov, M. Ballinger & A. Medeiros	65.39655	-164.6606
C	mucronata2	USA_AK_14c	U.S.A.	Alaska	Pond 10 near Taylor	D. J. Taylor, A. A. Kotov, M. Ballinger & A. Medeiros	65.39655	-164.6606
C	mucronata2	USA_AK_14d	U.S.A.	Alaska	Pond 10 near Taylor	D. J. Taylor, A. A. Kotov, M. Ballinger & A. Medeiros	65.39655	-164.6606
C	mucronata2	USA_AK_15a	U.S.A.	Alaska	Pond 7 near Taylor	D. J. Taylor, A. A. Kotov, M. Ballinger & A. Medeiros	65.4276	-164.6629
C	mucronata2	USA_AK_15b	U.S.A.	Alaska	Pond 7 near Taylor	D. J. Taylor, A. A. Kotov, M. Ballinger & A. Medeiros	65.4276	-164.6629
C	mucronata2	USA_AK_15c	U.S.A.	Alaska	Pond 7 near Taylor	D. J. Taylor, A. A. Kotov, M. Ballinger & A. Medeiros	65.4276	-164.6629
C	mucronata2	USA_AK_15d	U.S.A.	Alaska	Pond 7 near Taylor	D. J. Taylor, A. A. Kotov, M. Ballinger & A. Medeiros	65.4276	-164.6629

C	mucronata2	USA_AK_16a	U.S.A.	Alaska	Pond 8 near Taylor	D. J. Taylor, A. A. Kotov, M. Ballinger & A. Medeiros	65.4276	-164.6629
C	mucronata2	USA_AK_16b	U.S.A.	Alaska	Pond 8 near Taylor	D. J. Taylor, A. A. Kotov, M. Ballinger & A. Medeiros	65.4276	-164.6629
C	mucronata2	USA_AK_17a	U.S.A.	Alaska	Pond 3 near Taylor		65.4323	-164.6673
C	mucronata2	USA_AK_18a	U.S.A.	Alaska	Pond 1 near Taylor		n/a	n/a
C	mucronata2	USA_AK_19a	U.S.A.	Alaska	Margaret Lake, Kodiak		57.8304	-152.3601
C	mucronata2	USA_AK_19b	U.S.A.	Alaska	A pond, Fort Ambercrombie State Historical Park		57.7664	-152.4844
C	mucronata2	USA_AK_2a	U.S.A.	Alaska	Pong 17 near Nome	D. J. Taylor, A. A. Kotov, M. Ballinger & A. Medeiros	64.49532	-165.3728
C	mucronata2	USA_AK_2b	U.S.A.	Alaska	Pong 17 near Nome	D. J. Taylor, A. A. Kotov, M. Ballinger & A. Medeiros	64.49532	-165.3728
C	mucronata2	USA_AK_2c	U.S.A.	Alaska	Pong 17 near Nome	D. J. Taylor, A. A. Kotov, M. Ballinger & A. Medeiros	64.49532	-165.3728
C	mucronata2	USA_AK_2d	U.S.A.	Alaska	Pong 17 near Nome	D. J. Taylor, A. A. Kotov, M. Ballinger & A. Medeiros	64.49532	-165.3728
C	mucronata2	USA_AK_3a	U.S.A.	Alaska	Pond 2 near Nome	D. J. Taylor, A. A. Kotov, M. Ballinger & A. Medeiros	64.52882	-165.4173
C	mucronata2	USA_AK_3b	U.S.A.	Alaska	Pond 2 near Nome	D. J. Taylor, A. A. Kotov, M. Ballinger & A. Medeiros	64.52882	-165.4173
C	mucronata2	USA_AK_3c	U.S.A.	Alaska	Pond 2 near Nome	D. J. Taylor, A. A. Kotov, M. Ballinger & A. Medeiros	64.52882	-165.4173
C	mucronata2	USA_AK_3d	U.S.A.	Alaska	Pond 2 near Nome	D. J. Taylor, A. A. Kotov, M. Ballinger & A. Medeiros	64.52882	-165.4173
C	mucronata2	USA_AK_4a	U.S.A.	Alaska	Pond 2 near Nome	D. J. Taylor, A. A. Kotov, M. Ballinger & A. Medeiros	64.52882	-165.4173
C	mucronata2	USA_AK_4b	U.S.A.	Alaska	Glacial Lake	D. J. Taylor, A. A. Kotov, M. Ballinger & A. Medeiros	64.82653	-165.6725
C	mucronata2	USA_AK_6a	U.S.A.	Alaska	Pond 3 near Pilgrim	D. J. Taylor, A. A. Kotov, M. Ballinger & A. Medeiros	65.08134	-164.891
C	mucronata2	USA_AK_6b	U.S.A.	Alaska	Pond 3 near Pilgrim	D. J. Taylor, A. A. Kotov, M. Ballinger & A. Medeiros	65.08134	-164.891
C	mucronata2	USA_AK_6c	U.S.A.	Alaska	Pond 3 near Pilgrim	D. J. Taylor, A. A. Kotov, M. Ballinger & A. Medeiros	65.08134	-164.891
C	mucronata2	Mongolia_4c	Mongolia	Selenge Aimag	Affluent of the Orkhon River	D. P. Karabanov	50.24805	106.1406
C	mucronata2	Russia_Kamchatka_1a	Russia (Asian)	Kamchatka Area	Kronotskoe Lake	E. I. Izvekova	54.778	160.188
C	mucronata2	USA_AK_6d	U.S.A.	Alaska	Pond 3 near Pilgrim	D. J. Taylor, A. A. Kotov, M. Ballinger & A. Medeiros	65.08134	-164.891
C	mucronata2	USA_AK_7g	U.S.A.	Alaska	Pond 703 near Pilgrim	D. J. Taylor, A. A. Kotov, M. Ballinger & A. Medeiros	65.08895	-164.9228
C	mucronata2	USA_AK_7h	U.S.A.	Alaska	Pond 703 near Pilgrim	D. J. Taylor, A. A. Kotov, M. Ballinger & A. Medeiros	65.08895	-164.9228
C	mucronata2	Russia_Kamchatka_1b	Russia (Asian)	Kamchatka Area	Kronotskoe Lake	E. I. Izvekova	54.778	160.188
C	mucronata2	Russia_Yakutia_1a	Russia (Asian)	Yakutia Autonomous Republic	Lake 4 near the road Amga-Churapcha	А. А. Котов, А. И. Климовский	61.94688	132.6737
C	mucronata2	USA_AK_7i	U.S.A.	Alaska	Pond 703 near Pilgrim	D. J. Taylor, A. A. Kotov, M. Ballinger & A. Medeiros	65.08895	-164.9228
C	mucronata2	USA_AK_7j	U.S.A.	Alaska	Pond 703 near Pilgrim	D. J. Taylor, A. A. Kotov, M. Ballinger & A. Medeiros	65.08895	-164.9228
C	mucronata2	USA_AK_8a	U.S.A.	Alaska	Pond 2 near Pilgrim		65.0809	-164.8835
C	mucronata2	Russia_Tomsk_4a	Russia (Asian)	Tomsk Area	An oxbow water body of River Chulym 1	AA Kotov	57.8082	84.2458
C	mucronata2	Russia_Tomsk_4b	Russia (Asian)	Tomsk Area	An oxbow water body of River Chulym 1	AA Kotov	57.8082	84.2458
C	mucronata2	Russia_Khakassia_1a	Russia (Asian)	Khakass Autonomous Republic	Oxbow near Izykhskie Kopi, Abakan River	D. E. Shcherbakov	53.55582	91.27282
C	mucronata2	Russia_Kamchatka_1c	Russia (Asian)	Kamchatka Area	Kronotskoe Lake	E. I. Izvekova	54.778	160.188
C	mucronata2	Russia_Yakutia_7a	Russia (Asian)	Yakutia Autonomous Republic	Lake 1 on Melnikova Island, the Lena River	A. A. Kotov	63.85302	127.4713
D	mucronata4	Russia_Yakutia_10b	Russia (Asian)	Yakutia Autonomous Republic	A oxbow lake, River Khandyga, right bank of the Aldan River	A. A. Kotov	63.112	134.0446
D	mucronata4	Russia_Yakutia_2a	Russia (Asian)	Yakutia Autonomous Republic	A small lake near Lake Bolshaya Chabyda	А. А. Котов, А. И. Климовский	61.98367	129.3848
D	mucronata4	Russia_Yakutia_6a	Russia (Asian)	Yakutia Autonomous Republic	Lake Atyrdiakh, oxbow of the Handyga River	А. А. Котов	63.08686	134.0543
D	mucronata4	Russia_Primorsky_2a	Russia (Asian)	Primorski Territory	Iyinskoe Lake 4, region of Khanka Lake	N. M. Korovchinsky	44.92478	131.9617

D	mucronata4	Russia_Yakutia_6b	Russia (Asian)	Yakutia Autonomous Republic	Lake Atyrdiakh, oxbow of the Handyga River	А. А. Котов	63.08686	134.0543
D	mucronata4	Russia_Primorsky_2b	Russia (Asian)	Primorski Territory	Iyinskoe Lake 4, region of Khanka Lake	N. M. Korovchinsky	44.92478	131.9617
D	mucronata4	Russia_Yakutia_8d	Russia (Asian)	Yakutia Autonomous Republic	Lake 3 (Kvadratnoe) on Mel'nikov Island, the Lena River	A. A. Kotov	63.85395	127.4678
E	Scapoleberis cf. microcephala	USA_AK_5a	U.S.A.	Alaska	A pond near Council		64.8678	-163.6923
E	Scapholeberis cf. microcephala	Russia_Sakhalin_5a	Russia (Asian)	Sakhalin Area	A roadside ditch near Sovetskoe	A. A. Kotov & N. M. Korovchinsky	47.4557	142.7342
E	Scapholeberis cf. microcephala	Russia_Sakhalin_6a	Russia (Asian)	Sakhalin Area	Rvy, Sphagnum mine lake 1 (fishless) near Pokrovka	A. A. Kotov & N. M. Korovchinsky	47.32056	142.7072
E	Scapholeberis cf. microcephala	Russia_Sakhalin_7a	Russia (Asian)	Sakhalin Area	Rvy, Sphagnum mine lake 2 (with fish) near Pokrovka	A. A. Kotov & N. M. Korovchinsky	47.32058	142.7072
E	Scapholeberis cf. microcephala	Russia_Sakhalin_7b	Russia (Asian)	Sakhalin Area	Rvy, Sphagnum mine lake 2 (with fish) near Pokrovka	A. A. Kotov & N. M. Korovchinsky	47.32058	142.7072
F	rammneri1	Russia_Yakutia_1b	Russia (Asian)	Yakutia Autonomous Republic	Lake 4 near the road Amga-Churapcha	А. А. Котов, А. И. Климовский	61.94688	132.6737
F	rammneri1	Russia_YamaloNenets_1b	Russia (Asian)	Yamalo-Nenets Autonomour Area	Un-named lake 1 in Nadymsky Gorodok, 31 km from Obskaya Guba	A. B. Savinetsky	66.0602	72.00614
F	rammneri1	Mongolia_4a	Mongolia	Selenge Aimag	Affluent of the Orkhon River	D. P. Karabanov	50.24805	106.1406
F	rammneri1	Russia_Yakutia_4a	Russia (Asian)	Yakutia Autonomous Republic	Kuria Strelka, town of Yakutsk	А. А. Котов, А. И. Климовский	62.02006	129.7602
F	rammneri1	Russia_Yakutia_5a	Russia (Asian)	Yakutia Autonomous Republic	A roadside lake near Chepara willage Alagarsky Nasleg	A. A. Kotov	62.21303	131.4142
F	rammneri1	Russia_Yakutia_5b	Russia (Asian)	Yakutia Autonomous Republic	A roadside lake near Chepara willage Alagarsky Nasleg	A. A. Kotov	62.21303	131.4142
F	rammneri1	Russia_Yakutia_5c	Russia (Asian)	Yakutia Autonomous Republic	A roadside lake near Chepara willage Alagarsky Nasleg	A. A. Kotov	62.21303	131.4142
F	rammneri1	Russia_YamaloNenets_1c	Russia (Asian)	Yamalo-Nenets Autonomour Area	Un-named lake 1 in Nadymsky Gorodok, 31 km from Obskaya Guba	A. B. Savinetsky	66.0602	72.00614
F	rammneri1	Czechia_1c	Czech Republic		Pond in Pazderna	A. Petrusek	49.7166	18.4569
F	rammneri1	Czechia_1f	Czech Republic		Pond in Pazderna	A. Petrusek	49.7166	18.4569
F	rammneri1	Czechia_1d	Czech Republic		Pond in Pazderna	A. Petrusek	49.7166	18.4569
F	rammneri1	Czechia_1e	Czech Republic		Pond in Pazderna	A. Petrusek	49.7166	18.4569
F	rammneri1	Belgium_1h	Belgium		Nieuw Donk, Overmere-Berlare	K. Van Damme	51.03703	3.981533
F	rammneri1	Hungary_1a	Hungary		Lake Balaton		46.8	17.7
F	rammneri1	Hungary_1b	Hungary		Lake Balaton		46.8	17.7
F	rammneri1	Mongolia_1a	Mongolia	Zavkhan Aimag	A small puddle near Bayan Nuur (Lake)	A. A. Kotov	48.45144	95.17455
F	rammneri1	Russia_Tuva_1a	Russia (Asian)	Tuva Autonomous Republic	Puddles in the Mogen-Buren River valley	L. E. Savinetskaya & A. V. Tchabovsky	50.14244	89.82584
F	rammneri1	Russia_Tuva_1b	Russia (Asian)	Tuva Autonomous Republic	Puddles in the Mogen-Buren River valley	L. E. Savinetskaya & A. V. Tchabovsky	50.14244	89.82584
F	rammneri1	Russia_Tuva_1c	Russia (Asian)	Tuva Autonomous Republic	Puddles in the Mogen-Buren River valley	L. E. Savinetskaya & A. V. Tchabovsky	50.14244	89.82584
F	rammneri1	Russia_Tuva_1d	Russia (Asian)	Tuva Autonomous Republic	Puddles in the Mogen-Buren River valley	L. E. Savinetskaya & A. V. Tchabovsky	50.14244	89.82584
F	rammneri1	Russia_Tymen_1a	Russia (Asian)	Tyumen Area	Lake Maloe Setoe neas settlement of Kazanskoe	A. A. Kotov	55.62428	69.2087
F	rammneri1	Russia_YamaloNenets_3b	Russia (Asian)	Yamalo-Nenets Autonomour Area	Lake 2 in Nadymsky Gorodok, 31 km from Obskaya Guba	A. B. Savinetsky	66.2208	72.04506
F	rammneri1	Russia_Astrakhan_1a	Russia (European)	Astrakhan Area	An oxbow lake between Akhtuba and Volga	Y. R. Galimov	47.02222	47.59889
F	rammneri1	Mongolia_3b	Mongolia	Bayankhongor Aimag	Affluent of the Tuin-Gol River near town of Bayankhongor	D. P. Karabanov	46.30889	100.7464
F	rammneri1	Mongolia_1c	Mongolia	Zavkhan Aimag	A small puddle near Bayan Nuur (Lake)	A. A. Kotov	48.45144	95.17455
F	rammneri1	Mongolia_1b	Mongolia	Zavkhan Aimag	A small puddle near Bayan Nuur (Lake)	A. A. Kotov	48.45144	95.17455
G	rammneri2	Russia_Yakutia_2b	Russia (Asian)	Yakutia Autonomous Republic	A small lake near Lake Bolshaya Chabyda	А. А. Котов, А. И. Климовский	61.98367	129.3848
G	rammneri2	Russia_Primorsky_1a	Russia (Asian)	Primorski Territory	25. A puddle on the road to Maloe Utinoe Lake	N. M. Korovchinsky	43.4127	131.8214
G	rammneri2	Russia_Irkutsk_1a	Russia (Asian)	Irkutsk Area	A swamp, Olkhon Island, Baikal Lake	A. Chabovsky & L. Savinetskaya	53.07791	107.0001
G	rammneri2	Russia_Irkutsk_1b	Russia (Asian)	Irkutsk Area	A swamp, Olkhon Island, Baikal Lake	A. Chabovsky & L. Savinetskaya	53.07791	107.0001
G	rammneri2	Russia_Chita_1a	Russia (Asian)	Chita Area	Mine lakes near Chernovskaya station	D. Vasilenko	51.97	113.23
G	rammneri2	Russia_Chita_1b	Russia (Asian)	Chita Area	Mine lakes near Chernovskaya station	D. Vasilenko	51.97	113.23
G	rammneri2	Russia_Chita_1c	Russia (Asian)	Chita Area	Mine lakes near Chernovskaya station	D. Vasilenko	51.97	113.23
G	rammneri2	Russia_Chita_1d	Russia (Asian)	Chita Area	Mine lakes near Chernovskaya station	D. Vasilenko	51.97	113.23
G	rammneri2	Russia_Chita_1e	Russia (Asian)	Chita Area	Mine lakes near Chernovskaya station	D. Vasilenko	51.97	113.23
G	rammneri2	Russia_Kamchatka_1d	Russia (Asian)	Kamchatka Area	Kronotskoe Lake	E. I. Izvekova	54.778	160.188
H	rammneri3	USA_NY_1a	U.S.A.	New York	Lake Erie		42.73	-78.94
H	rammneri3	Canada_BC_1a	Canada	British Columbia	Port Hardy	S.J. Connelly	50.6889	-127.4321
H	rammneri3	Canada_BC_2a	Canada	British Columbia	A pond 3 near Woss	S.J. Connelly	50.2605	-126.8167
H	rammneri3	Canada_BC_3a	Canada	British Columbia	Lake 2 near Woss	S.J. Connelly	50.2172	-126.5741
H	rammneri3	Canada_BC_4a	Canada	British Columbia	Hoomak Lake	S.J. Connelly	50.2086	-126.5205
H	rammneri3	Canada_BC_5a	Canada	British Columbia	Rooney	S.J. Connelly	50.3571	-126.1557
H	rammneri3	Canada_BC_6a	Canada	British Columbia	Beaver Lake in British Columbia	S.J. Connelly	50.1522	-125.6236
H	rammneri3	Canada_BC_7a	Canada	British Columbia	Roberts	S.J. Connelly	50.2162	-125.5444
H	rammneri3	Canada_BC_8a	Canada	British Columbia	Reed	S.J. Connelly	50.1324	-125.2796

H	rammneri3	Canada_BC_9a	Canada	British Columbia	Dickson Lake	S.J. Connelly	49.3964	-125.0798
H	rammneri3	Canada_BC_10a	Canada	British Columbia	Moran Pond	S.J. Connelly	49.3725	-125.0447
H	rammneri3	Canada_BC_11a	Canada	British Columbia	Sproat Lake	S.J. Connelly	49.2923	-124.9293
H	rammneri3	Canada_BC_12a	Canada	British Columbia	Darlington Lake	S.J. Connelly	48.9646	-124.7201
H	rammneri3	Canada_BC_13a	Canada	British Columbia	Brennan Lake	S.J. Connelly	49.2144	-124.0499
H	rammneri3	Canada_BC_14a	Canada	British Columbia	Long Lake	S.J. Connelly	49.2094	-124.0203
H	rammneri3	Canada_BC_15a	Canada	British Columbia	Somenos	S.J. Connelly	48.8037	-123.7089
H	rammneri3	Canada_BC_16a	Canada	British Columbia	Quamichian Lake	S.J. Connelly	48.7885	-123.6713
H	rammneri3	Canada_BC_17a	Canada	British Columbia	Lake Shawnigan	S.J. Connelly	48.6389	-123.6332
H	rammneri3	Canada_BC_18a	Canada	British Columbia	St. Mary Lake	S.J. Connelly	48.8943	-123.5371
H	rammneri3	Canada_BC_19a	Canada	British Columbia	Langford Lake	S.J. Connelly	48.4443	-123.5269
H	rammneri3	Canada_BC_20a	Canada	British Columbia	Blackburn Lake	S.J. Connelly	48.8217	-123.4837
H	rammneri3	Canada_BC_21a	Canada	British Columbia	Stowell Lake	S.J. Connelly	48.7822	-123.4447
H	rammneri3	Canada_BC_22a	Canada	British Columbia	Elk Lake	S.J. Connelly	48.5333	-123.4
H	rammneri3	Canada_BC_23a	Canada	British Columbia	Beaver Lake, Victoria	S.J. Connelly	48.5102	-123.3907
H	rammneri3	Argentina_1a	Argentina		humic pond	KS Costanzo, MB Balling, DJ Taylor	-51.0191	-71.7777
H	rammneri3	Argentina_1b	Argentina		humic pond	KS Costanzo, MB Balling, DJ Taylor	-51.0191	-71.7777
H	rammneri3	Argentina_1c	Argentina		humic pond	KS Costanzo, MB Balling, DJ Taylor	-51.0191	-71.7777
H	rammneri3	Argentina_1d	Argentina		humic pond	KS Costanzo, MB Balling, DJ Taylor	-51.0191	-71.7777
H	rammneri3	Argentina_2a	Argentina		shallow roadside pond	KS Costanzo, MB Balling, DJ Taylor	-48.7615	-70.2551
H	rammneri3	Argentina_2b	Argentina		shallow roadside pond	KS Costanzo, MB Balling, DJ Taylor	-48.7615	-70.2551
H	rammneri3	Argentina_2c	Argentina		shallow roadside pond	KS Costanzo, MB Balling, DJ Taylor	-48.7615	-70.2551
H	rammneri3	Argentina_3a	Argentina		pond with much vegetation	KS Costanzo, MB Balling, DJ Taylor	-48.7522	-70.2734
H	rammneri3	Argentina_3b	Argentina		pond with much vegetation	KS Costanzo, MB Balling, DJ Taylor	-48.7522	-70.2734
H	rammneri3	Argentina_3c	Argentina		pond with much vegetation	KS Costanzo, MB Balling, DJ Taylor	-48.7522	-70.2734
H	rammneri3	Argentina_3d	Argentina		pond with much vegetation	KS Costanzo, MB Balling, DJ Taylor	-48.7522	-70.2734
H	rammneri3	Argentina_3e	Argentina		pond with much vegetation	KS Costanzo, MB Balling, DJ Taylor	-48.7522	-70.2734
H	rammneri3	Argentina_3f	Argentina		pond with much vegetation	KS Costanzo, MB Balling, DJ Taylor	-48.7522	-70.2734
H	rammneri3	Argentina_3g	Argentina		pond with much vegetation	KS Costanzo, MB Balling, DJ Taylor	-48.7522	-70.2734
H	rammneri3	Argentina_3h	Argentina		pond with much vegetation	KS Costanzo, MB Balling, DJ Taylor	-48.7522	-70.2734
H	rammneri3	Argentina_3i	Argentina		pond with much vegetation	KS Costanzo, MB Balling, DJ Taylor	-48.7522	-70.2734
H	rammneri3	Argentina_3j	Argentina		pond with much vegetation	KS Costanzo, MB Balling, DJ Taylor	-48.7522	-70.2734
H	rammneri3	Argentina_3k	Argentina		pond with much vegetation	KS Costanzo, MB Balling, DJ Taylor	-48.7522	-70.2734
H	rammneri3	Argentina_4a	Argentina		small roadside pond	KS Costanzo, MB Balling, DJ Taylor	-45.936	-71.5852
H	rammneri3	Argentina_4b	Argentina		small roadside pond	KS Costanzo, MB Balling, DJ Taylor	-45.936	-71.5852
H	rammneri3	Chile_5a	Chile	Décima Primera Región de Aisén del General Carlos Ibáñez del Campo	small lake, large littoral zone	KS Costanzo, MB Balling, DJ Taylor	-44.6467	-72.3258
H	rammneri3	Chile_5b	Chile	Décima Primera Región de Aisén del General Carlos Ibáñez del Campo	small lake, large littoral zone	KS Costanzo, MB Balling, DJ Taylor	-44.6467	-72.3258
H	rammneri3	Argentina_4c	Argentina		small roadside pond	KS Costanzo, MB Balling, DJ Taylor	-45.936	-71.5852
H	rammneri3	Argentina_4d	Argentina		small roadside pond	KS Costanzo, MB Balling, DJ Taylor	-45.936	-71.5852
H	rammneri3	Canada_BC_24a	Canada	British Columbia	Swan Lake	S.J. Connelly	48.4644	-123.3732
H	rammneri3	Canada_BC_25a	Canada	British Columbia	Bhorm Lake	S.J. Connelly	49.8218	-123.1335
H	rammneri3	Canada_BC_26a	Canada	British Columbia	Daisy Swamp	S.J. Connelly	50.0667	-123.0998
H	rammneri3	Canada_BC_27a	Canada	British Columbia	One Mile Lake	S.J. Connelly	50.3121	-122.8062
H	rammneri3	Canada_BC_28a	Canada	British Columbia	Lower Jeoffrey	S.J. Connelly	50.3651	-122.5059
H	rammneri3	USA_PA_1a	U.S.A.	Pennsylvania	Indian Pond	S.J. Connelly	41.2689	-76.9318
H	rammneri3	USA_NY_2a	U.S.A.	New York	Bryant Woods Pond	DJ Taylor	43.0211	-78.7858
H	rammneri3	Canada_BC_29a	Canada	British Columbia	Portage Bay	S.J. Connelly	47.6495	-122.3123
H	rammneri3	USA_NY_6a	U.S.A.	New York	Seneca Lake	S.J. Connelly	46.6742	-76.9037
H	rammneri3	USA_MI_1b	U.S.A.	Michigan	A pond near Sister Lakes	DJ Taylor	42.28	-83.79
H	rammneri3	USA_MI_1a	U.S.A.	Michigan	A pond near Sister Lakes	DJ Taylor	42.28	-83.79
H	rammneri3	Canada_BC_30a	Canada	British Columbia	Union Bay		47.6486	-122.2991
H	rammneri3	Canada_BC_31a	Canada	British Columbia	Kawkawa Lake	S.J. Connelly	49.3857	-121.3935
H	rammneri3	USA_NM_1a	U.S.A.	New Mexico	Mattox Lale	A.A. Kotov & W.H. Piel	32.7181	-103.3075
H	rammneri3	USA_NY_3a	U.S.A.	New York	Beaver Lake	DJ Taylor	43.12	-76.48
H	rammneri3	USA_MN_1a	U.S.A.	Minnesota			43.0211	-78.7858
H	rammneri3	USA_NY_1b	U.S.A.	New York	Lake Erie		42.73	-78.94
H	rammneri3	USA_PA_1b	U.S.A.	Pennsylvania	Indian Pond		41.2689	-76.9318
H	rammneri3	USA_NY_4a	U.S.A.	New York	Round Pond		43.018983	-77.563455
H	rammneri3	Canada_ON_1a	Canada	Ontario	Grenadier Pond		43.6421	-79.4675
H	rammneri3	Canada_ON_2a	Canada	Ontario	Jordan wetland	S.J. Connelly	43.1575	-79.3772
H	rammneri3	Canada_ON_3a	Canada	Ontario	Jordan Harbor	S.J. Connelly	43.1581	-79.3736

H	rammneri3	Canada_ON_4a	Canada	Ontario	Jourban Inlet	S.J. Connelly	43.1581	-79.3736
H	rammneri3	Canada_BC_32a	Canada	British Columbia	Strathoone Lake	S.J. Connelly		
H	rammneri3	USA_MA_1a	U.S.A.	Massachusetts	Walden Pond	A.A. Kotov, W.H. Piel	42.4397	-70.3347
H	rammneri3	USA_CT_1a	U.S.A.	Connecticut	Pine Acres Lake	D.J. Taylor	41.7833	-72.0841
H	rammneri3	Chile_5c	Chile	Décima Primera Región de Aisén del General Carlos Ibáñez del Campo	small lake, large littoral zone	KS Costanzo, MB Balling, DJ Taylor	-44.6467	-72.3258
H	rammneri3	Canada_NWF_1a	Canada	New Foundland	A pond near Wiltondale	L.J. Hovind, M. Faustova	49.4585	-57.5389
H	rammneri3	Chile_5d	Chile	Décima Primera Región de Aisén del General Carlos Ibáñez del Campo	small lake, large littoral zone	KS Costanzo, MB Balling, DJ Taylor	-44.6467	-72.3258
H	rammneri3	Chile_6a	Chile	Décima Región de Los Lagos	Lake Lonconao	KS Costanzo, MB Balling, DJ Taylor	-43.2405	-71.9287
H	rammneri3	Chile_6b	Chile	Décima Región de Los Lagos	Lake Lonconao	KS Costanzo, MB Balling, DJ Taylor	-43.2405	-71.9287
H	rammneri3	Chile_6c	Chile	Décima Región de Los Lagos	Lake Lonconao	KS Costanzo, MB Balling, DJ Taylor	-43.2405	-71.9287
H	rammneri3	Chile_6d	Chile	Décima Región de Los Lagos	Lake Lonconao	KS Costanzo, MB Balling, DJ Taylor	-43.2405	-71.9287
H	rammneri3	Chile_7a	Chile	Décima Región de Los Lagos	small lake in Futaleufú	KS Costanzo, MB Balling, DJ Taylor	-43.1846	-71.8602
H	rammneri3	Chile_7b	Chile	Décima Región de Los Lagos	small lake in Futaleufú	KS Costanzo, MB Balling, DJ Taylor	-43.1846	-71.8602
H	rammneri3	Chile_7c	Chile	Décima Región de Los Lagos	small lake in Futaleufú	KS Costanzo, MB Balling, DJ Taylor	-43.1846	-71.8602
H	rammneri3	Chile_7d	Chile	Décima Región de Los Lagos	small lake in Futaleufú	KS Costanzo, MB Balling, DJ Taylor	-43.1846	-71.8602
H	rammneri3	Chile_7e	Chile	Décima Región de Los Lagos	small lake in Futaleufú	KS Costanzo, MB Balling, DJ Taylor	-43.1846	-71.8602
H	rammneri3	Chile_7f	Chile	Décima Región de Los Lagos	small lake in Futaleufú	KS Costanzo, MB Balling, DJ Taylor	-43.1846	-71.8602
H	rammneri3	Chile_7g	Chile	Décima Región de Los Lagos	small lake in Futaleufú	KS Costanzo, MB Balling, DJ Taylor	-43.1846	-71.8602
H	rammneri3	Chile_7h	Chile	Décima Región de Los Lagos	small lake in Futaleufú	KS Costanzo, MB Balling, DJ Taylor	-43.1846	-71.8602
H	rammneri3	USA_MA_1b	U.S.A.	Massachusetts	Walden Pond	A.A. Kotov, W.H. Piel	42.4397	-70.3347
H	Scapholeberis cf. mucronata	contig c29081_g2_i1	U.S.A.	Massachusetts		doi: 10.1098/ rspb.2018.1524.		
I	rammneri4	Chile_8a	Chile	Décima Primera Región de Aisén del General Carlos Ibáñez del Campo	Lake Elizalde	K. S. Costanzo, M. B. Balling, D. J. Taylor	-45.775	-72.1753
J	Scapholeberis freyi	Canada_ON_5a	Canada	Ontario	Bracebridge		45.04	-79.31
J	Scapholeberis freyi	USA_SC_1a	U.S.A.	South Carolina	Congri Swamp		33.82849	-80.82443
J	Scapholeberis freyi	USA_SC_2a	U.S.A.	South Carolina	Bamberg	DJ Taylor, HL Sprenger, AR Omilian	33.2167	-80.9887
J	Scapholeberis "mucronata group"	ENA: LS991522.1	Belgium			doi: 10.1016/ j.ympev.2019.05.018.		
K	Scapholeberis kingii	Australia_NSW_1a	Australia	New South Wales	Farm Dam	S. Jacobs & Y. Kobayashi	-36.38507	149.9764
K	Scapholeberis kingii	Australia_NSW_1b	Australia	New South Wales	Farm Dam	S. Jacobs & Y. Kobayashi	-36.38507	149.9764
K	Scapholeberis kingii	Australia_NSW_1c	Australia	New South Wales	Farm Dam	S. Jacobs & Y. Kobayashi	-36.38507	149.9764
K	Scapholeberis kingii	Australia_NSW_1d	Australia	New South Wales	Farm Dam	S. Jacobs & Y. Kobayashi	-36.38507	149.9764
K	Scapholeberis kingii	Australia_NSW_1e	Australia	New South Wales	Farm Dam	S. Jacobs & Y. Kobayashi	-36.38507	149.9764
L	Scapholeberis cf. kingii	Japan_1a	Japan	Wakayama	HigashiIke	S Ishida	34.1899	135.239
L	Scapholeberis cf. kingii	Japan_1b	Japan	Wakayama	HigashiIke	S Ishida	34.1899	135.239
L	Scapholeberis cf. kingii	Japan_2a	Japan	Wakayama	Shinike, Iwade County	S Ishida	34.2808	135.3297
L	Scapholeberis cf. kingii	Japan_2b	Japan	Wakayama	Shinike, Iwade County	S Ishida	34.2808	135.3297
L	Scapholeberis cf. kingii	Japan_2i	Japan	Wakayama	Shinike, Iwade County	S Ishida	34.2808	135.3297
L	Scapholeberis cf. kingii	Japan_2c	Japan	Wakayama	Shinike, Iwade County	S Ishida	34.2808	135.3297
L	Scapholeberis cf. kingii	Japan_2d	Japan	Wakayama	Shinike, Iwade County	S Ishida	34.2808	135.3297
L	Scapholeberis cf. kingii	Japan_2e	Japan	Wakayama	Small Pond, Katsuragi	S Ishida	34.3055	135.5261
L	Scapholeberis cf. kingii	Japan_2f	Japan	Wakayama	Small Pond, Katsuragi	S Ishida	34.3055	135.5261
L	Scapholeberis cf. kingii	Japan_2g	Japan	Wakayama	Small Pond, Katsuragi	S Ishida	34.3055	135.5261
L	Scapholeberis cf. kingii	Japan_2h	Japan	Wakayama	Small Pond, Katsuragi	S Ishida	34.3055	135.5261
L	Scapholeberis cf. kingii	Japan_3b	Japan	Hiroshima	Pond, Ashida	S Ishida	34.5167	133.2881
L	Scapholeberis cf. kingii	Japan_3a	Japan	Hiroshima	Pond, Ashida	S Ishida	34.5167	133.2881
L	Scapholeberis cf. kingii	Russia_Primorsky_3a	Russia (Asian)	Primorski Territory	Puddle 2 near the reservoir of Luchegorskaya power station	A. A. Kotov & S. A. Ivanov	46.45208	134.2991
L	Scapholeberis cf. kingii	Russia_Primorsky_3b	Russia (Asian)	Primorski Territory	Puddle 2 near the reservoir of Luchegorskaya power station	A. A. Kotov & S. A. Ivanov	46.45208	134.2991
L	Scapholeberis cf. kingii	Japan_4a	Japan	Kyoto	Midorogaike	S Ishida	35.0547	135.7711
L	Scapholeberis cf. kingii	Japan_4b	Japan	Kyoto	Midorogaike	S Ishida	35.0547	135.7711
L	Scapholeberis cf. kingii	Russia_Primorsky_4a	Russia (Asian)	Primorski Territory	A puddle, Partizanskaya Street 1, village of Fedoseevka	A. A. Kotov & S. A. Ivanov	46.53117	134.2777
L	Scapholeberis cf. kingii	Japan_4c	Japan	Kyoto	Midorogaike	S Ishida	35.0547	135.7711
L	Scapholeberis cf. kingii	Japan_4d	Japan	Kyoto	Midorogaike	S Ishida	35.0547	135.7711
L	Scapholeberis cf. kingii	Japan_5a	Japan	Shiga	Nishinoko	S Ishida	35.1573	136.0956
L	Scapholeberis cf. kingii	Japan_5b	Japan	Shiga	Nishinoko	S Ishida	35.1573	136.0956

L	Scapholeberis cf. kingii	Russia_Sakhalin_1a	Russia (Asian)	Sakhalin Area	Puddles between see and road, Puzina Peninsula	A. A. Kotov & N. M. Korovchinsky	46.80519	143.271
L	Scapholeberis cf. kingii	Russia_Sakhalin_2a	Russia (Asian)	Sakhalin Area	A pyddle in the River Susuya valley, Yuzhno-Sakhalinsk	A. A. Kotov & N. M. Korovchinsky	46.95055	142.7023
L	Scapholeberis cf. kingii	Russia_Sakhalin_3a	Russia (Asian)	Sakhalin Area	Puddle near Susuya River, Yuzhno-Sakhalinsk	A. A. Kotov & N. M. Korovchinsky	46.94725	142.6948
L	Scapholeberis cf. kingii	Russia_Sakhalin_4a	Russia (Asian)	Sakhalin Area	Puddle near Pokrovka	A. A. Kotov & N. M. Korovchinsky	47.3223	142.7053
L	Scapholeberis cf. kingii	Japan_6b	Japan	Shiga	Shojyo Ko	S Ishida	35.4456	135.9836
L	Scapholeberis cf. kingii	Japan_6a	Japan	Shiga	Shojyo Ko	S Ishida	35.4456	135.9836
L	Scapholeberis cf. kingii	Japan_7c	Japan	Yamagata	Ohnuma	S Ishida	38.2447	140.2038
L	Scapholeberis cf. kingii	Japan_7b	Japan	Yamagata	Ohnuma	S Ishida	38.2447	140.2038
L	Scapholeberis cf. kingii	Japan_7a	Japan	Yamagata	Ohnuma	S Ishida	38.2447	140.2038
L	Scapholeberis cf. kingii	Russia_Adygeya_1a	Russia (European)	The Republic of Adygea	A canal to rice paddies	E. I. Bekker	44.94389	39.0555
L	Scapholeberis cf. kingii	Russia_Adygeya_1b	Russia (European)	The Republic of Adygea	A canal to rice paddies	E. I. Bekker	44.94389	39.0555
L	Scapholeberis cf. kingii	Japan_7d	Japan	Yamagata	Ohnuma	S Ishida	38.2447	140.2038
L	Scapholeberis cf. kingii	Russia_Sakhalin_1b	Russia (Asian)	Sakhalin Area	Puddles between see and road, Puzina Peninsula	A. A. Kotov & N. M. Korovchinsky	46.80519	143.271
L	Scapholeberis cf. kingii	Russia_Sakhalin_1c	Russia (Asian)	Sakhalin Area	Puddles between see and road, Puzina Peninsula	A. A. Kotov & N. M. Korovchinsky	46.80519	143.271
L	Scapholeberis cf. kingii	Russia_Sakhalin_1d	Russia (Asian)	Sakhalin Area	Puddles between see and road, Puzina Peninsula	A. A. Kotov & N. M. Korovchinsky	46.80519	143.271
L	Scapholeberis cf. kingii	Russia_Sakhalin_3b	Russia (Asian)	Sakhalin Area	Puddle near Susuya River, Yuzhno-Sakhalinsk	A. A. Kotov & N. M. Korovchinsky	46.94725	142.6948
M	Scapholeberis spinifera	Chile_3b	Chile	Décima Segunda Región de Magallanes y La Antártica Chilena	A shallow drainage ditch	KS Costanzo, MB Balling, DJ Taylor	-51.8831	-72.1089
M	Scapholeberis spinifera	Chile_3c	Chile	Décima Segunda Región de Magallanes y La Antártica Chilena	A shallow drainage ditch	KS Costanzo, MB Balling, DJ Taylor	-51.8831	-72.1089
M	Scapholeberis spinifera	Chile_4a	Chile	Décima Segunda Región de Magallanes y La Antártica Chilena	A ditch, no littoral zone	KS Costanzo, MB Balling, DJ Taylor	-51.7066	-72.4475
M	Scapholeberis spinifera	Chile_4b	Chile	Décima Segunda Región de Magallanes y La Antártica Chilena	A ditch, no littoral zone	KS Costanzo, MB Balling, DJ Taylor	-51.7066	-72.4475
M	Scapholeberis spinifera	Chile_4c	Chile	Décima Segunda Región de Magallanes y La Antártica Chilena	A ditch, no littoral zone	KS Costanzo, MB Balling, DJ Taylor	-51.7066	-72.4475
M	Scapholeberis spinifera	Chile_4d	Chile	Décima Segunda Región de Magallanes y La Antártica Chilena	A ditch, no littoral zone	KS Costanzo, MB Balling, DJ Taylor	-51.7066	-72.4475
M	Scapholeberis spinifera	Chile_1a	Chile	Décima Segunda Región de Magallanes y La Antártica Chilena	A roadside pond, road to Fuerte Bulnes	KS Costanzo, MB Balling, DJ Taylor	-53.36715	-70.97501
M	Scapholeberis spinifera	Argentina_2a	Argentina	Décima Segunda Región de Magallanes y La Antártica Chilena	Parque Sarmiento pond	KS Costanzo, MB Balling, DJ Taylor	-33.105	-64.3339
M	Scapholeberis spinifera	Argentina_2b	Argentina	Décima Segunda Región de Magallanes y La Antártica Chilena	Parque Sarmiento pond	KS Costanzo, MB Balling, DJ Taylor	-33.105	-64.3339
M	Scapholeberis spinifera	Argentina_2c	Argentina	Décima Segunda Región de Magallanes y La Antártica Chilena	Parque Sarmiento pond	KS Costanzo, MB Balling, DJ Taylor	-33.105	-64.3339
M	Scapholeberis spinifera	Argentina_2d	Argentina	Décima Segunda Región de Magallanes y La Antártica Chilena	Parque Sarmiento pond	KS Costanzo, MB Balling, DJ Taylor	-33.105	-64.3339
M	Scapholeberis spinifera	Argentina_2e	Argentina	Décima Segunda Región de Magallanes y La Antártica Chilena	Parque Sarmiento pond	KS Costanzo, MB Balling, DJ Taylor	-33.105	-64.3339
M	Scapholeberis spinifera	Argentina_2f	Argentina	Décima Segunda Región de Magallanes y La Antártica Chilena	Parque Sarmiento pond	KS Costanzo, MB Balling, DJ Taylor	-33.105	-64.3339
M	Scapholeberis spinifera	Chile_3a	Chile	Décima Segunda Región de Magallanes y La Antártica Chilena	A shallow drainage ditch	KS Costanzo, MB Balling, DJ Taylor	-51.8831	-72.1089
N	Scapholeberis armata	USA_MA_2a	U.S.A.	Massachusetts	Fresh Pond		41.6851	-70.1497
N	Scapholeberis armata	USA_MA_2b	U.S.A.	Massachusetts	Fresh Pond		41.6851	-70.1497
O	Megafenestra aurita	Ukraine_1a	Ukraine	Odessa Area	A flooded area near Tiligul River near Beriozovka	E.I. Bekker	47.173	30.9214
O	Megafenestra aurita	Ukraine_1b	Ukraine	Odessa Area	A flooded area near Tiligul River near Beriozovka	E.I. Bekker	47.173	30.9214
O	Megafenestra aurita	Ukraine_2a	Ukraine	Odessa Area	A pond near Tiligul River near Beriozovka	E.I. Bekker	47.173	30.9215
O	Megafenestra aurita	Ukraine_1c	Ukraine	Odessa Area	A flooded area near Tiligul River near Beriozovka	E.I. Bekker	47.173	30.9214
O	Megafenestra aurita	Ukraine_1d	Ukraine	Odessa Area	A flooded area near Tiligul River near Beriozovka	E.I. Bekker	47.173	30.9214
O	Megafenestra aurita	Ukraine_1e	Ukraine	Odessa Area	A flooded area near Tiligul River near Beriozovka	E.I. Bekker	47.173	30.9214
O	Megafenestra aurita	Ukraine_2b	Ukraine	Odessa Area	A pond near Tiligul River near Beriozovka	E.I. Bekker	47.173	30.9215
P	Megafenestra sp.	Russia_Yakutia_10a	Russia (Asian)	Yakutia Autonomous Republic	A oxbow lake, River Khandyga, right bank of the Aldan River	A. A. Kotov	63.112	134.0446
P	Megafenestra sp.	USA_AK_1a	U.S.A.	Alaska	Birch Lake, Anchorage	D. J. Taylor, A. A. Kotov, M. Ballinger & A. Medeiros	61.14558	-149.9384
P	Megafenestra sp.	USA_AK_1b	U.S.A.	Alaska	Birch Lake, Anchorage	D. J. Taylor, A. A. Kotov, M. Ballinger & A. Medeiros	61.14558	-149.9384

P	Megafenestra sp.	USA_AK_1c	U.S.A.	Alaska	Birch Lake, Anchorage	D. J. Taylor, A. A. Kotov, M. Ballinger & A. Medeiros	61.14558	-149.9384
P	Megafenestra sp.	USA_AK_1d	U.S.A.	Alaska	Birch Lake, Anchorage	D. J. Taylor, A. A. Kotov, M. Ballinger & A. Medeiros	61.14558	-149.9384
P	Megafenestra sp.	USA_AK_7a	U.S.A.	Alaska	Pond 703 near Pilgrim	D. J. Taylor, A. A. Kotov, M. Ballinger & A. Medeiros	65.08895	-164.9228
P	Megafenestra sp.	USA_AK_7b	U.S.A.	Alaska	Pond 703 near Pilgrim	D. J. Taylor, A. A. Kotov, M. Ballinger & A. Medeiros	65.08895	-164.9228
P	Megafenestra sp.	USA_AK_7c	U.S.A.	Alaska	Pond 703 near Pilgrim	D. J. Taylor, A. A. Kotov, M. Ballinger & A. Medeiros	65.08895	-164.9228
P	Megafenestra sp.	USA_AK_7d	U.S.A.	Alaska	Pond 703 near Pilgrim	D. J. Taylor, A. A. Kotov, M. Ballinger & A. Medeiros	65.08895	-164.9228
P	Megafenestra sp.	USA_AK_7e	U.S.A.	Alaska	Pond 703 near Pilgrim	D. J. Taylor, A. A. Kotov, M. Ballinger & A. Medeiros	65.08895	-164.9228
P	Megafenestra sp.	USA_AK_7f	U.S.A.	Alaska	Pond 703 near Pilgrim	D. J. Taylor, A. A. Kotov, M. Ballinger & A. Medeiros	65.08895	-164.9228
Q	Megafenestra nasuta	USA_NY_5a	U.S.A.	New York	Jordan wetland	S.J. Connelly	43.1575	-79.3772
outgroup	Diaphanosoma dubium	NC_037488.1	China		Liuxihe reservoir	doi: 10.1080/23802359.2017.1413295	39.97	116.2
UV	Ceriodaphnia reticulata	LS991488.1						
UV	Ceriodaphnia sp. 1	This study	U.S.A.					
UV	Ceriodaphnia sp. 2	This study	U.S.A.					
UV	Daphnia ambigua	This study	U.S.A.	NY	Deep Pond			
UV	Daphnia carinata	KP721459.1	China			doi: 10.1080/23802359.2016.1172045		
UV	Daphnia dentifera	This study	U.S.A.	NY	Round Pond			
UV	Daphnia dubia	This study	Canada	NFLD				
UV	Daphnia ephemeralis	This study	Canada	Ontario				
UV	Daphnia galeata	LC152879.1	Japan	Nagano	Lake Shirakaba	doi: 10.1016/j.gene.2017.02.019		
UV	Daphnia hispanica	LS991493.1	Spain					
UV	Daphnia laevis	MK059395.1	Brazil					
UV	Daphnia longicephala	This study	Australia		Fleurieu Peninsula			
UV	Daphnia magna	This study	U.S.A.	Nebraska	Cresent Lake			
UV	Daphnia occidentalis	This study	Australia	Western Australia	Northcliff			
UV	Daphnia pulex	AF117817.1	Canada	Ontario		doi: 10.1016/s0378-1119(99)00151-1		
UV	Daphnia truncata	This study	Australia	Western Australia				
UV	Moina brachiata	LS991521.1						
UV	Moina sp. 1	Contig: c24402_g3_i1	U.S.A.	Arizona		doi: 10.1098/rspb.2018.1524.		
UV	Moina sp. 2	This study	Mexico	Guanajuato	Pond, Valle de Santiago			
UV	Simocephalus sp. 1	This study	U.S.A.	Connecticut	Pine Acres Lake			
UV	Simocephalus sp. 2	This study	U.S.A.	Alaska	Nome pond			

Figure S1.

Maximum likelihood mitochondrial phylogeny of neustonic daphniids (*Scapholeberis* and *Megafenestra*) and known genera and subgenera of daphniids. Bold letters (A-Q) indicate geographic clades. Dots at the nodes indicate transfer bootstrap expectation/nonparametric bootstrap support (solid red is >95/>95, hollow red is >95/>80 and gray is >95/<80). The S. rammneri species group is shown by a green box and the *S. mucronata* species group by a pink box. The non-daphniid cladoceran, *Diaphanosoma dubium*, was used for outgroup rooting. The five sequences from public databases are indicated by accession numbers or contig number. All other sequences are from this study (Accessions listed in data availability).

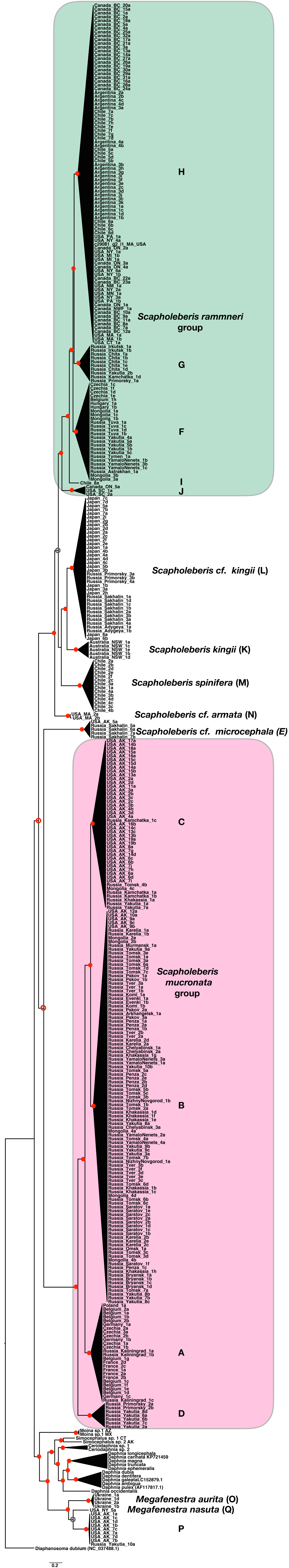
H
Scapholeberis rammneri
group
G
F
I
J
Scapholeberis cf. kingii (L)
Scapholeberis kingii (K)
Scapholeberis spinifera (M)
Scapholeberis cf. armata (N)
Scapholeberis cf. microcephala (E)
C
Scapholeberis
mucronata
group
B
A
D
Megafenestra aurita (O)
Megafenestra nasuta (Q)
P
0.2

Figure S2.

Expanded Maximum likelihood mitochondrial phylogeny of neustonic daphniids in the *Scapholeberis rammneri* clade. *S. spinifera* is used for outgroup rooting and the sister clade to *S. rammneri*, *S. freyi* (J) is also shown. Numbers at the nodes indicate approximate likelihood ratio tests (aLRTs)/nonparametric bootstrap support. Blue sequences are from public genome/transcriptome projects and red sequences are presumptive introductions to South America from western North America.

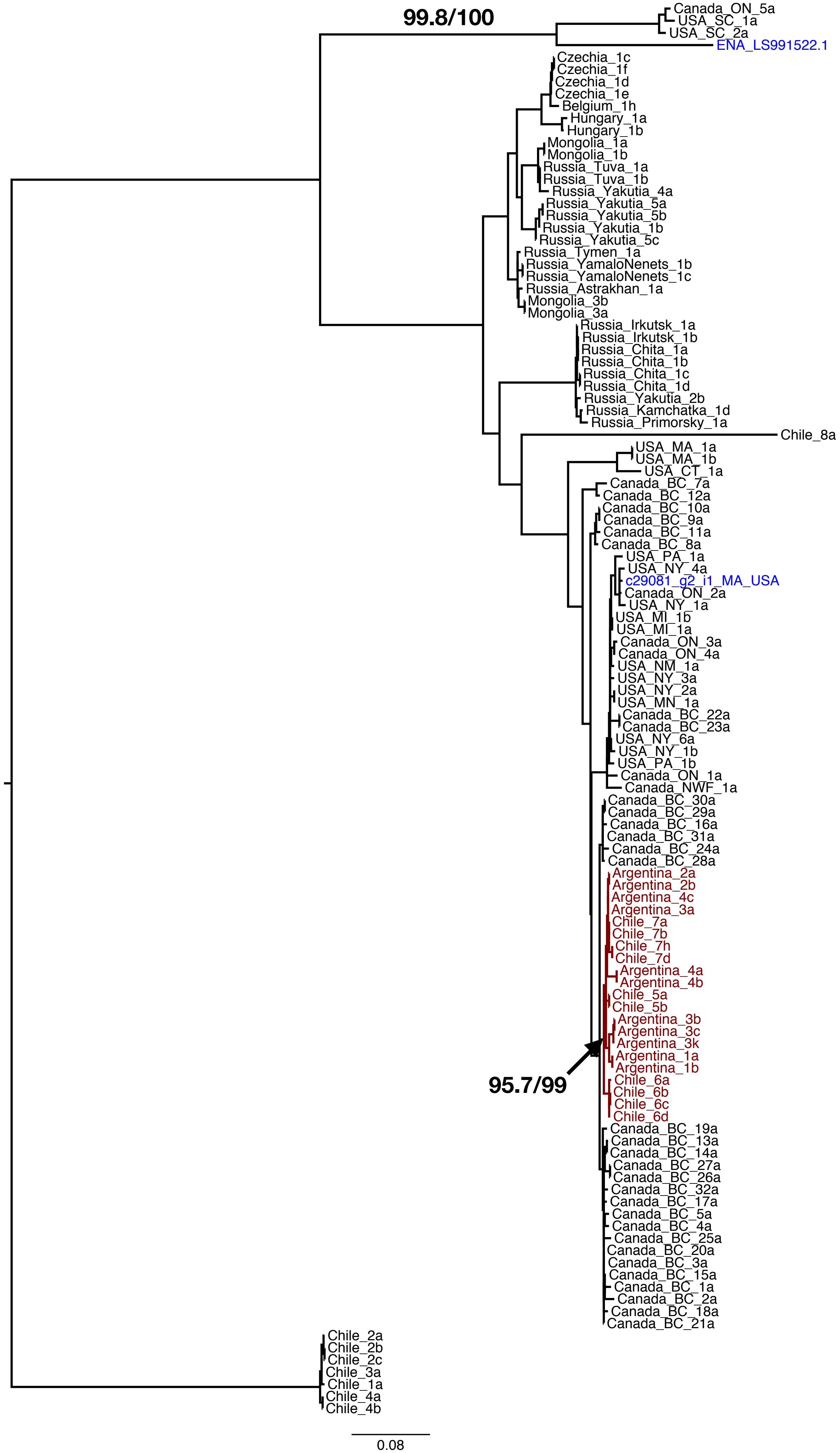
99.8/100
Canada_ON_5a
USA_SC_1a
USA_SC_2a
ENA_LS991522.1
Czechia_1c
Czechia_1f
Czechia_1d
Czechia_1e
Belgium_1h
Hungary_1a
Hungary_1b
Mongolia_1a
Mongolia_1b
Russia_Tuva_1a
Russia_Tuva_1b
Russia_Yakutia_4a
Russia_Yakutia_5a
Russia_Yakutia_5b
Russia_Yakutia_1b
Russia_Yakutia_5c
Russia_Tymen_1a
Russia_YamaloNenets_1b
Russia_YamaloNenets_1c
Russia_Astrakhan_1a
Mongolia_3b
Mongolia_3a
Russia_Irkutsk_1a
Russia_Irkutsk_1b
Russia_Chita_1a
Russia_Chita_1b
Russia_Chita_1c
Russia_Chita_1d
Russia_Yakutia_2b
Russia_Kamchatka_1d
Russia_Primorsky_1a
Chile_8a
USA_MA_1a
USA_MA_1b
USA_CT_1a
Canada_BC_7a
Canada_BC_12a
Canada_BC_10a
Canada_BC_9a
Canada_BC_11a
Canada_BC_8a
USA_PA_1a
USA_NY_4a
c29081_g2_i1_MA_USA
Canada_ON_2a
USA_NY_1a
USA_MI_1b
USA_MI_1a
Canada_ON_3a
Canada_ON_4a
USA_NM_1a
USA_NY_3a
USA_NY_2a
USA_MN_1a
Canada_BC_22a
Canada_BC_23a
USA_NY_6a
USA_NY_1b
USA_PA_1b
Canada_ON_1a
Canada_NWF_1a
Canada_BC_30a
Canada_BC_29a
Canada_BC_16a
Canada_BC_31a
Canada_BC_24a
Canada_BC_28a
Argentina_2a
Argentina_2b
Argentina_4c
Argentina_3a
Chile_7a
Chile_7b
Chile_7h
Chile_7d
Argentina_4a
Argentina_4b
Chile_5a
Chile_5b
Argentina_3b
Argentina_3c
Argentina_3k
Argentina_1a
Argentina_1b
Chile_6a
Chile_6b
Chile_6c
Chile_6d
95.7/99
Canada_BC_19a
Canada_BC_13a
Canada_BC_14a
Canada_BC_27a
Canada_BC_26a
Canada_BC_32a
Canada_BC_17a
Canada_BC_5a
Canada_BC_4a
Canada_BC_25a
Canada_BC_20a
Canada_BC_3a
Canada_BC_15a
Canada_BC_1a
Canada_BC_2a
Canada_BC_18a
Canada_BC_21a
Chile_2a
Chile_2b
Chile_2c
Chile_3a
Chile_1a
Chile_4a
Chile_4b
0.08

Figure S3.

Maximum likelihood mitochondrial phylogeny of neustonic daphniids (*Scapholeberis* and *Megafenestra*) and known genera and subgenera of daphniids estimated with a heterotachy model (GTR+FO*H4) in IQtree. Bold letters (A-Q) indicate geographic clades. The *S. rammneri* species group is shown by a green box and the *S. mucronata* species group by a pink box. The non-daphniid cladoceran, *Diaphanosoma dubia*, was used for outgroup rooting.

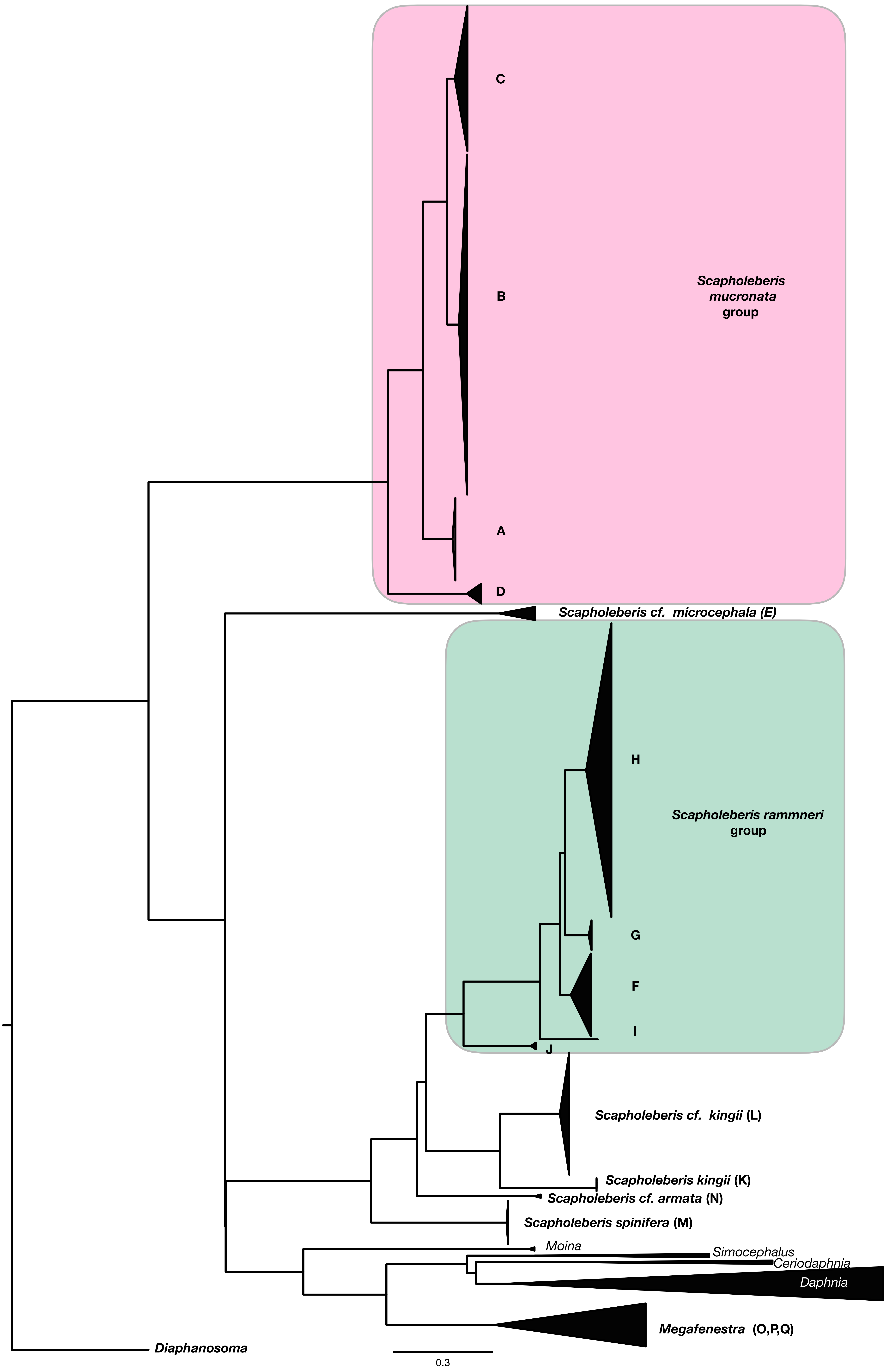
C
B
Scapholeberis mucronata group
A
D
Scapholeberis cf. microcephala (E)
H
Scapholeberis rammneri group
G
F
I
J
Scapholeberis cf. kingii (L)
Scapholeberis kingii (K)
Scapholeberis cf. armata (N)
Scapholeberis spinifera (M)
Moina
Simocephalus
Ceriodaphnia
Daphnia
Megafenestra (O,P,Q)
Diaphanosoma
0.3
